# STING activation induces polarized cytokine secretion of IFN-β and IL-17A promoting photoreceptor death and choroidal disruption in age-related macular degeneration

**DOI:** 10.1038/s41419-026-08491-w

**Published:** 2026-02-27

**Authors:** Chao Huang, Vishnu Suresh Babu, Sridhar Bammidi, Jakob N. Arnold, Martin Ebeling, Gabriella Widmer, Pamela Strassburger, Mirjana Lazendic, Sabine Grüner, Janis Koester, Puja Dutta, Stacey Hose, Sonali Singh, Pooja Gautam, Eleonora M. Lad, Peter D. Westenskow, Oksana Kutsyr, Karl G. Csaky, Sayan Ghosh, Srinivasa R. Sripathi, Alan D. Proia, Miguel Flores-Bellver, Debasish Sinha, Derrick Feenstra

**Affiliations:** 1 Roche Pharma Research and Early Development, Ophthalmology Discovery, Roche Innovation Center Basel, F. Hoffmann-La Roche Ltd., Basel, Switzerland; 2 Wilmer Eye Institute, The Johns Hopkins University School of Medicine, Baltimore, MD USA; 3 Henderson Ocular Stem Cell Laboratory, Retina Foundation of the Southwest, Dallas, TX USA; 4 Department of Ophthalmology, Duke University Medical Center, Durham, NC USA; 5 CellSight Ocular Stem Cell and Regeneration Research Program, Department of Ophthalmology, Sue Anschutz-Rodgers Eye Center, University of Colorado Anschutz Medical Campus, Aurora, CO USA; 6 Department of Ophthalmology, University of Texas Southwestern Medical Center, Texas Dallas, USA; 7 Department of Pathology, Duke University Medical Center, Durham, NC USA; 8 Department of Pathology, Campbell University Jerry M. Wallace School of Osteopathic Medicine, Lillington, NC USA

**Keywords:** Diseases, Cell biology, Inflammation

## Abstract

Age-related macular degeneration (AMD) represents one of the therapeutic challenges of aging eye diseases. Our investigation reveals the stimulator of interferon genes (STING) pathway as an orchestrator of immune-mediated retinal degeneration, exhibiting biphasic, stage-dependent functionality—providing cytoprotection in healthy tissue but driving pathogenic inflammation during early AMD progression. Through immunohistochemical analysis of human eyes, we demonstrate stage-dependent cytoplasmic STING upregulation with parallel IFN-β activation. Using patient-derived induced pluripotent stem cells-retinal pigment epithelium (iPSC-RPE) from AMD siblings, we discovered polarized cytokine secretion: apical IFN-β triggers photoreceptor apoptosis in human retinal organoids, while basal IL-17A compromises choroidal neovascularization. The *Cryba1* conditional knockout (cKO) AMD-like mouse model confirms STING-driven IL-17A expression, while *Il17a* knock-in mice substantiate vascular alterations. STING activation establishes a pathogenic feed-forward loop between interferons and IL-17A. Single-cell transcriptomics following AAV2-mediated IFN-β overexpression reveals metabolic and phototransduction dysregulation. Both pharmacological STING inhibition with SN-011 and genetic approaches demonstrate therapeutic rescue. *Cryba1*/*Sting* double heterozygous (dhet) mice maintain homeostatic gene expression preserving retinal architecture and function. These findings establish STING as the master regulator simultaneously controlling multiple AMD pathologies through spatially organized inflammation, transforming from protective surveillance to pathogenic driver, and identifying a unified therapeutic target with demonstrated functional rescue across multiple experimental paradigms.

## Introduction

Age-related macular degeneration (AMD) represents an unparalleled public health challenge, affecting more patients than all invasive cancers combined and more than double the number of those with Alzheimer’s disease [1, 2]. The most severe form, geographic atrophy (GA), is characterized by progressive, irreversible loss of retinal pigment epithelium (RPE) and photoreceptors in the central retina, leading to profound visual impairment that renders patients unable to read, recognize faces, or maintain independence [3]. Despite its enormous clinical burden [4] and the urgent need for therapeutic interventions [5], the molecular mechanisms driving AMD progression remain incompletely understood [6, 7], and effective treatments remain elusive [8, 9]. Central to AMD pathogenesis is chronic inflammation within the retinal microenvironment [10]. Mounting evidence indicates that innate immune activation plays a critical role in disease initiation and progression [11–13], with complement system dysregulation [14, 15], inflammasome activation [16], and aberrant cytokine signaling [17, 18] all implicated in retinal degeneration. However, the upstream molecular switches that coordinate these inflammatory cascades and determine the spatial specificity of retinal damage characteristic of AMD remain poorly defined.

The stimulator of interferon genes (STING) pathway has emerged as a master regulator of innate immunity, serving as a cytosolic DNA sensor that triggers robust type I interferon responses upon detection of microbial or self-derived nucleic acids [19, 20]. Under physiological conditions, STING activation provides essential antimicrobial defense and cellular stress responses [21–23]. However, dysregulated STING signaling has been increasingly recognized as a driver of pathogenic inflammation in aging-related diseases [24, 25], autoimmune disorders [26, 27], and tissue degeneration [28]. In the context of neurodegeneration, sustained STING activation promotes inflammatory responses that contribute to neuronal dysfunction and death [25, 29], suggesting potential relevance to retinal degenerative diseases.

While STING’s role in systemic inflammatory diseases is well-established [30], its function in retinal homeostasis and disease has remained largely unexplored. The retina presents a unique anatomical environment where the RPE forms a critical blood-retinal barrier, maintaining distinct apical and basal compartments that interface with photoreceptors and choroidal vasculature, respectively [31]. This polarized architecture is essential for normal visual function [32], yet how inflammatory signaling pathways like STING operate within this specialized context, and whether they contribute to the characteristic pattern of retinal damage observed in AMD, has not been investigated. Furthermore, the mechanisms underlying the selective vulnerability of specific retinal cell types [33, 34] and regions [35] in AMD remain poorly understood. GA exhibits a distinctive pattern of damage, with photoreceptor death occurring in defined areas while adjacent regions remain initially preserved [36]. Simultaneously, choroidal vascular dysfunction develops independently but coordinately with neurosensory retinal changes [37]. This spatially organized pattern of degeneration suggests the existence of precisely regulated pathogenic mechanisms, yet the molecular basis for such selective vulnerability has not been identified.

Here, we report the finding of STING as a central orchestrator of retinal inflammation in AMD, revealing a previously unrecognized mechanism that coordinates both photoreceptor death and choroidal dysfunction through spatially organized inflammatory signaling. We demonstrate that STING undergoes stage-dependent activation in human AMD eyes, transitioning from protective immune surveillance in healthy tissue [19] to pathogenic inflammation during disease progression. Most importantly, we identify a novel mechanism of polarized cytokine secretion from AMD patient-derived RPE cells [31], whereby STING activation simultaneously triggers apical interferon-β (IFN-β) release that directly damages photoreceptors and basal interleukin-17A (IL-17A) secretion that compromises choroidal vasculature. Our findings establish STING as the first identified master regulator capable of explaining the coordinated yet spatially distinct patterns of retinal damage characteristic of GA [38]. Through comprehensive analysis using patient-derived cells, animal models, and therapeutic interventions, we demonstrate that STING activation creates self-perpetuating inflammatory loops involving both type I and type II interferons that sustain chronic retinal inflammation. These findings provide fundamental insights into AMD pathogenesis and identify STING inhibition as a promising therapeutic strategy that could simultaneously address multiple pathogenic processes underlying this debilitating disease.

## Results

### Stage-dependent STING expression and IFN-β activation in human AMD

**Fig. 1:**
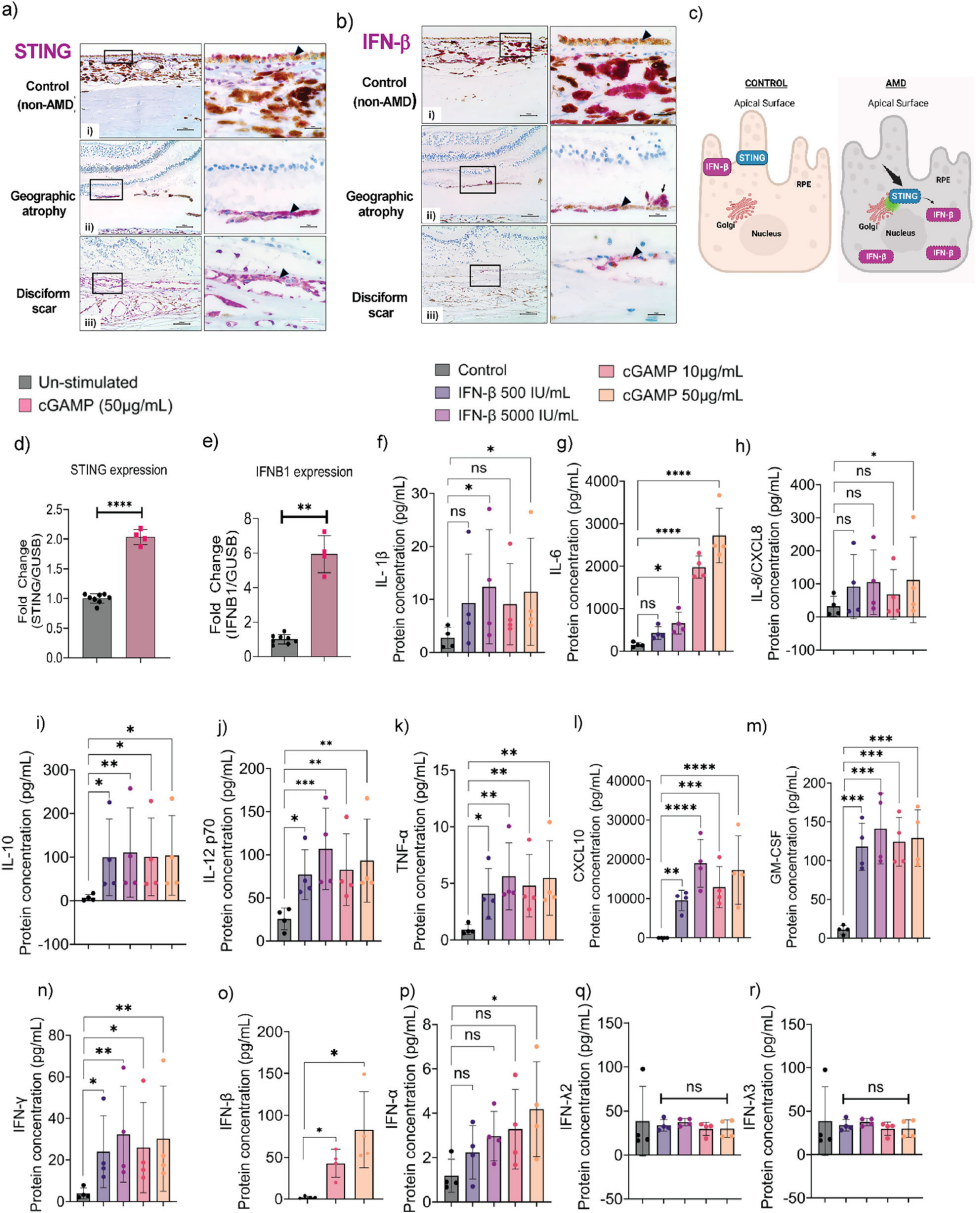
STING and IFN-β proteins are expressed in the RPE of human retinal tissues and upregulated in response to cGAMP stimulation. Representative immunohistochemistry micrographs showing (**a**) STING and **b** IFN-β expression in macular RPE from non-AMD controls (87-year-old black man), GA (77-year-old black woman), and disciform scar (88-year-old white woman) subjects. **a** i) STING protein (purple) was mainly expressed in the apical side of macular RPE cells in non-AMD control eyes. STING was also expressed in choroidal vascular endothelial cells, inflammatory cells, and stromal cells. The neurosensory retina is absent in the sections due to formalin-fixation artifactual retinal detachment. ii) STING protein tended to be diffusely localized within the cytosol of macular RPE cells at the periphery of GA, creating increased staining intensity. iii) STING was maximally expressed in the RPE of eyes with disciform scars, which exhibited RPE duplication. The area within boxes in the photomicrographs on the left are shown at higher magnification in the photomicrographs to their right; arrowheads indicate the RPE. **b** i) Ten of eleven non-AMD control eyes had no detectable IFN-β in the macular RPE. Choroidal stromal cells and melanocytes usually expressed IFN-β, sometimes strongly, as in this eye. ii) Six of eleven eyes had focal or patchy expression of IFN-β in RPE cells at the periphery of GA. Three eyes with GA and four with disciform scars also had subretinal melanin-laden cells (arrow) expressing IFN-β. iii) Four of eight eyes had focal or patchy IFN−β expression in duplicated RPE within disciform scars. The area within boxes in the photomicrographs on the left is shown at higher magnification in the photomicrographs to their right; arrowheads indicate the RPE. Scale bar = 100 μm, and for higher magnification the scale bars = 25 μm. *n* = 11. **c** Schematic showing STING and IFN-β localization in non-AMD control and AMD RPE tissues. Stimulation of primary human RPE with cGAMP (50 μg/mL) for 12 h increased transcriptional levels of (**d**) STING and **e** IFN-β, as measured by qPCR. Luminex assay shows that cGAMP (50 μg/mL) and IFN-β (5000 IU/mL) induced production of proinflammatory and antiviral cytokines: **f** IL-1β, **g** IL-6, **h** IL-8/CXCL8, **i** IL-10, **j** IL-12, **k** TNF-α, **l** CXCL10, **m** GM-CSF, **n** IFN-γ, **o** IFN-β (without IFN-β induction), and **p** IFN-α. No increases were detected for **q** IFN-λ2 and **r** IFN-λ3. Values represent the mean ± s.d. from four independent experiments. Statistical analysis was performed using two-way ANOVA with Dunnett’s multiple comparisons test. ns not significant. *p < 0.05, **p < 0.01, ***p < 0.001, ****p < 0.0001.

Our immunohistochemical analysis of human donor eyes at different stages of AMD progression revealed increased STING expression. In non-AMD controls, STING was predominantly localized to the apical surface of macular RPE cells (Fig. 1a, panel i, Fig. 1c). However, STING exhibited a diffuse cytoplasmic distribution in AMD macular RPE cells, with expression levels increasing across disease stages from nominal but elevated expression in GA to its maximal expression in eyes with disciform scars [39] (flat, circular scars that form beneath the neurosensory retina during the late stages of AMD; Fig. 1a, panels ii-iii, Fig. 1c).

While transient IFN-β activation by STING provides cytoprotection against viral infections and cellular stress [21, 23], sustained STING–IFN-β signaling contributes to pathogenic inflammation and mitochondrial dysfunction [40, 41]. Our analysis revealed that IFN-β immunoreactivity in macular RPE cells closely paralleled STING expression patterns—minimal in control RPE (Fig. 1b, panel i, Fig. 1c) but showing focal and patchy positivity in GA and disciform scar tissues (Fig. 1b, panels ii-iii, Fig. 1c).

Functional studies in primary human RPE cells treated with the STING agonist cyclic guanosine monophosphate–adenosine monophosphate (cGAMP) [42] demonstrated that STING activation induced a robust inflammatory response, significantly increasing STING (Fig. 1d) and IFN-β (Fig. 1e) transcriptional levels. Moreover, treatment with IFN-β or cGAMP in human RPE cell cultures promoted the secretion of proinflammatory cytokines [43], including IL-1β (Fig. 1f), IL-6 (Fig. 1g), IL-8/CXCL8 (Fig. 1h), IL-10 (Fig. 1i), IL-12 (Fig. 1j), TNF-α (Fig. 1k), CXCL10 (Fig. 1l), and GM-CSF (Fig. 1m).

Notably, treatment of human RPE cell cultures with cGAMP or IFN-β also induced IFN-γ secretion (Fig. 1n), indicating potential crosstalk between type I and type II interferon responses [44]. IFN-β levels were significantly increased upon cGAMP stimulation and exceeded the quantifiable range upon IFN-β stimulation (Fig. 1o). We found significant upregulation of IFN-α upon cGAMP treatment, but not with IFN-β exposure (Fig. 1p). However, IFN-λ2 (Fig. 1q) and IFN-λ3 (Fig. 1r) levels remained largely unaffected, suggesting a selective proinflammatory response driven by IFN-β.

### Polarized cytokine secretion from patient-derived iPSC-RPE cells

**Fig. 2:**
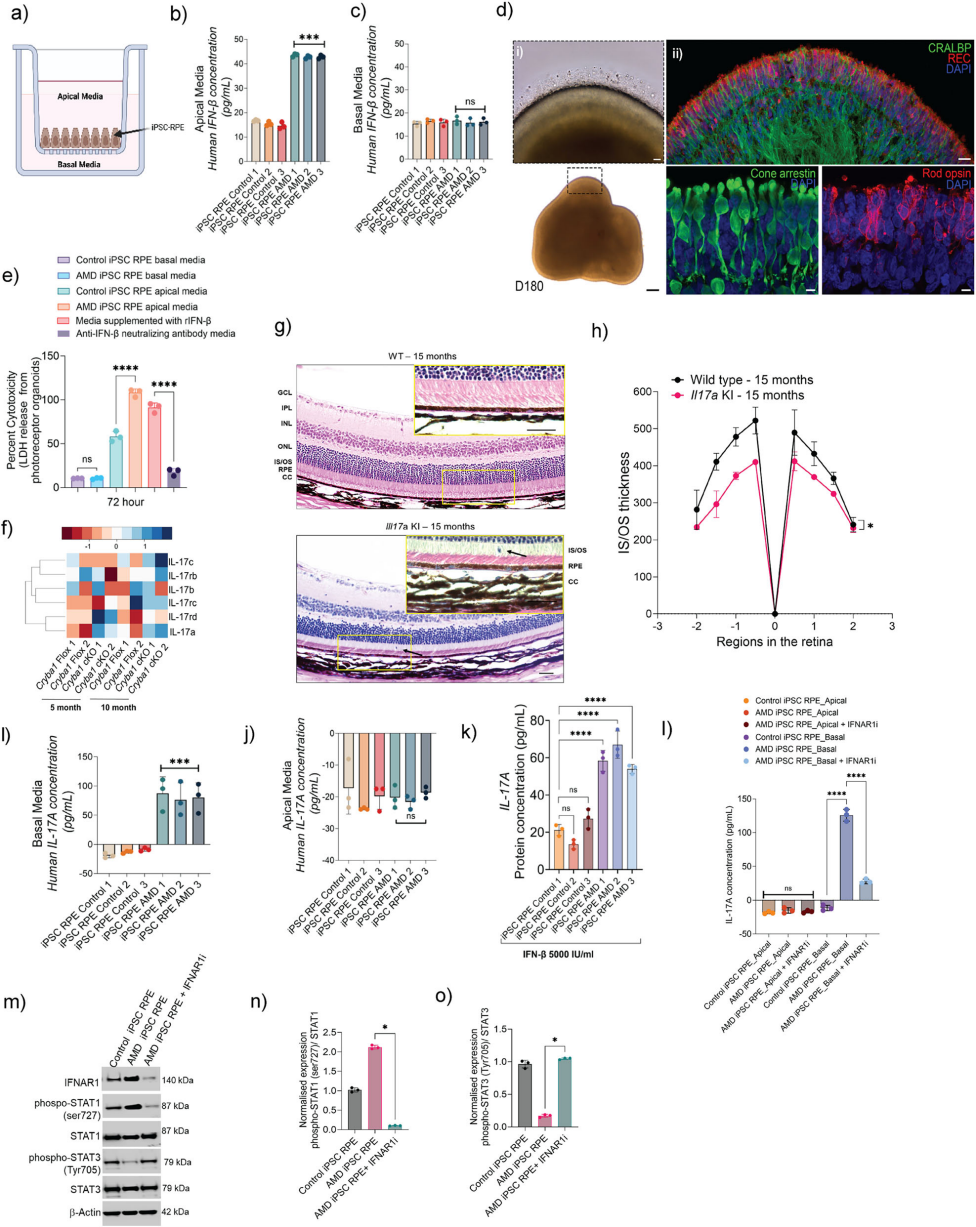
Polarized secretion of IFN-β from RPE contributes to photoreceptor and retinal cell damage. **a** Schematic of polarized human iPSC-RPE culture in a transwell system. Medium from the insert (upper chamber) was collected as apical media, and media from the bottom chamber as basal media. IFN-β levels in (**b**) apical and (**c**) basal media of iPSC-RPE derived from AMD patients (*n* = 3) and their healthy siblings as controls (*n* = 3). **d** Human iPSC-derived three-dimensional retinal organoid at day 180 (D180) of differentiation showing i) structure (brightfield microscopy) and ii) expression of Müller glial marker CRALBP (Green) and photoreceptor markers recoverin (Red), cone arrestin (Green), and rod opsin (Red). **e** LDH release assay showing the percentage of cytotoxicity in retinal organoids exposed to media derived from iPSC-derived control (*n* = 3) and AMD (*n* = 3) RPE cultures. **f** Heatmap showing differential expression of IL-17 pathway genes in RPE from 5- and 10-month-old *Cryba1* floxed and *Cryba1* cKO mice. IL-17A levels are significantly higher in *Cryba1* cKO mice at both time points compared to controls. **g** H&E staining of retinal sections reveal choroidal and retinal abnormalities in *Il17a* KI mice. *Il17a* KI animals differed from WT mice by having marked choroidal thickening with an increased number of melanocytes (lower panel), vacuolated RPE cells (arrows, lower panel), and mild thinning of photoreceptor inner and outer segments (*n* = 3). Scale bar= 50 μm (zoomed inset: 20 μm). **h** Spider plot showing the IS/OS layer thickness across the entire retina Secreted IL-17A levels measured by ELISA in (**i**) basal and (**j**) apical media of polarized iPSC RPE cultures, as well as (**k**) 24-h IFN-β treatment (5000 IU/mL) to iPSC-RPE from AMD subjects (*n* = 3) and non-AMD controls (*n* = 3). **l** Secreted IL-17A was measured by ELISA after IFNAR1 inhibition using anifrolumab (100 nM for 24 h) in the apical and basal media of AMD iPSC-derived RPE cells, with no-treatment sibling control iPSC RPE cells used as controls. **m** western blot analysis was conducted to assess the expression levels of IFNAR1, phospho-STAT1, and phospho-STAT3, along with total STAT1 and STAT3, following IFNAR1 inhibition in AMD iPSC RPE cells. Densitometry analysis (*n* = 3 AMD iPSC RPE) quantified the levels of (**n**) phospho-STAT1 and (**o**) phospho-STAT3 upon IFNAR1 inhibition. Values represent mean ± s.d. from triplicate experiments (*n* = 3). Abbreviations: REC recoverin, DAPI 4′,6-diamidino-2-phenylindole, CC3 cleaved caspase-3, IS/OS inner/outer segment of photoreceptors, ONL outer nuclear layer, OPL outer plexiform layer, INL inner nuclear layer. Scale bars: 200 μm (**d**-i), 20 μm (**d**-ii). Statistical significance was determined using one-way ANOVA with Tukey’s multiple comparisons test. ns not significant. ***p < 0.001, ****p < 0.0001.

A critical mechanistic insight emerged from our investigation using induced pluripotent stem cells-retinal pigmented epithelium (iPSC-RPE) derived from patients (harboring *CFH* Y402H variant) with intermediate to advanced stages of dry AMD whose eyes had macular pigmentary changes associated with hyperreflective foci (HRF) overlying drusen and from their unaffected siblings [45]. These RPE cells were cultured on a porous membrane within a transwell system to simulate the physiological RPE barrier [46], enabling comparative analysis of AMD-specific molecular and functional differences across apical and basal surfaces (Fig. 2a).

The iPSC-RPE cells derived from AMD patients with *CFH* Y402H variant exhibited significantly elevated secretion of IFN-β from the apical surface (Fig. 2b), which interfaces with photoreceptors, while basal secretion of IFN-β remained unaltered (Fig. 2c). This polarized cytokine release offers a mechanistic explanation for the selective vulnerability of photoreceptors observed in AMD progression. Indeed, photoreceptor apoptosis has been observed at the margins of GA in patients with advanced non-neovascular AMD [47]. To further examine the contribution of IFN-β to the development of GA, we exposed 180-day-old (D180) human iPSC-derived retinal organoids [48] to conditioned media from the apical and basal compartments of iPSC-RPE cultures. Retinal organoids provide a valuable model for investigating the human retina, identifying disease mechanisms, and facilitating the discovery of new treatments [48]. By D180, the inner and outer retinal layers are clearly defined and contain the precursors of the major retinal cell types, including rod and cone photoreceptor cells (Fig. 2d) that respond to light.

Apical media from AMD-derived RPE cells induced significant photoreceptor cell death as evident from lactate dehydrogenase (LDH) release assays conducted at 72 h, which revealed significantly elevated LDH levels in retinal organoids exposed to either apical media from iPSC-RPE cells of AMD subjects with *CFH* Y402H variant or media supplemented with recombinant IFN-β (Fig. 2e). We also observed increased cell death in retinal organoids exposed to apical media from iPSC-RPE AMD cultures compared to those from no AMD RPE controls as evidenced by TUNEL assay, cleaved caspase-3 staining, and pyroptotic cleaved caspase-1 expression (Fig. S1a–d). These results suggest a potential connection between the apically polarized secretion of IFN-β by AMD iPSC-RPE cells and subsequent photoreceptor degeneration, providing important mechanistic insight into how damage progresses in the neurosensory retina during AMD.

### *Cryba1* cKO model demonstrates STING-driven IL-17A expression

Building on these findings of polarized IFN-β secretion in human AMD cells, we sought to validate and expand our understanding through an established animal model. The *Cryba1* (encoding βA3/A1-crystallin) conditional knockout (cKO) mouse represents a valuable AMD-like model characterized by fundamental impairments in RPE lysosomal function [49]. These impairments trigger epigenetic changes through reduced histone deacetylase 3 (HDAC3) activity, while simultaneously disrupting both autophagy and phagocytosis in the RPE [50], which together initiate a cascade of pathological changes, including drusen-like deposits, lipofuscin accumulation, and melanosome retention [51–53].

Loss of *Cryba1* relieves its inhibitory control on STING, resulting in STING upregulation [53] and elevated IFN-β production. Consistent with this, *Cryba1* cKO RPE exhibited heightened STING expression and a transcriptomic profile enriched for chronic inflammatory pathways, including significant upregulation of *Il17a* (Fig. 2f). IL-17A exhibits context-dependent functionality, serving protective roles in epithelial homeostasis under physiological conditions but varying across tissues and disease states [54, 55]. Chronic IL-17A overexpression in *Il17a* knock-in (KI) mice resulted in choroidal thickening, RPE cytoplasmic vacuoles, thinning of photoreceptor inner and outer segments, and subtle infiltration of immune-like cells (Figs. 2g, h and S1e). This indicates a shift in IL-17A function from homeostatic to pathogenic, driven by chronic stimuli like STING-mediated IFN-β production.

Mechanistically, previous studies showed that IL-17A compromises the blood-retinal barrier by altering tight junction proteins and increasing vascular permeability [56], activates endothelial cells by upregulating adhesion molecules (ICAM-1, VCAM-1) [57], induces VEGF-A expression in RPE cells and infiltrating macrophages [58], and triggers complement activation through increased C3 expression and MAC deposition [59]. Collectively, these effects compromise choroidal vasculature and the RPE–Bruch’s membrane interface, hallmarks of AMD pathology and therefore positions the STING–IFN-β–IL-17A axis as a key pathological driver in the aging retina.

STING-induced IFN-β signaling, in turn, amplifies IL-17A expression establishing a previously unknown pro-inflammatory feedback loop that sustains retinal inflammation. In addition to directly enhancing IL-17A expression, STING-induced IFN-β signaling also promotes the induction of IFN-γ (Fig. 1o), a well-established upstream regulator of IL-17A, thereby establishing a feed-forward inflammatory loop involving type I and II interferons and IL-17A [60].

We next evaluated IL-17A secretion from polarized iPSC-RPE cells and observed a pronounced enrichment of IL-17A release from the basal surface, which interfaces with the choroidal circulation (Fig. 2i), while apical secretion, which would impact the neurosensory retina, was unchanged [31] (Fig. 2j). Notably, treating iPSC-RPE cells with recombinant IFN-β increased IL-17A secretion compared to untreated controls. This effect was even more pronounced in iPSC-RPE cells derived from AMD patients with *CFH* Y402H variant, suggesting a direct regulatory relationship between these factors (Fig. 2k). To investigate the mechanism behind the preferential basal secretion of IL-17A and its relationship with the apical secretion of IFN-β mediated by STING activation in RPE, we treated polarized iPSC RPE cells from both control and AMD subjects in transwell cultures with the IFNAR1 inhibitor anifrolumab (100 nM) for 24 h. ELISA analysis revealed a significant reduction in IL-17A levels upon blocking IFNAR1 receptors (Fig. 2l). This reduction signifies the dependency of IL-17A production on the IFN-β signaling pathway, highlighting a autocrine mechanism in the context of RPE cell function. We further investigated the mechanism underlying the STING/IFN-β/IL-17A loop by blocking IFNAR1 with anifrolumab (100 nM) in iPSC-RPE cells from AMD subjects. Under basal conditions, STING-driven IFN-β production activates IFNAR1, leading to STAT1 phosphorylation and up-regulation of IL-17A, which reinforces inflammation through an autocrine loop. Inhibiting IFNAR1 markedly reduces STAT1 phosphorylation (Fig. 2m, n), thereby weakening this STAT1–IL-17A feed-forward cycle. Loss of dominant STAT1 signaling simultaneously relieves its suppressive effect on STAT3 [61], resulting in increased STAT3 phosphorylation (Fig. 2m, o) and a shift toward a less inflammatory, more repair-oriented signaling profile.

### Consequences of 10 week sustained IFN-β signaling

**Fig. 3:**
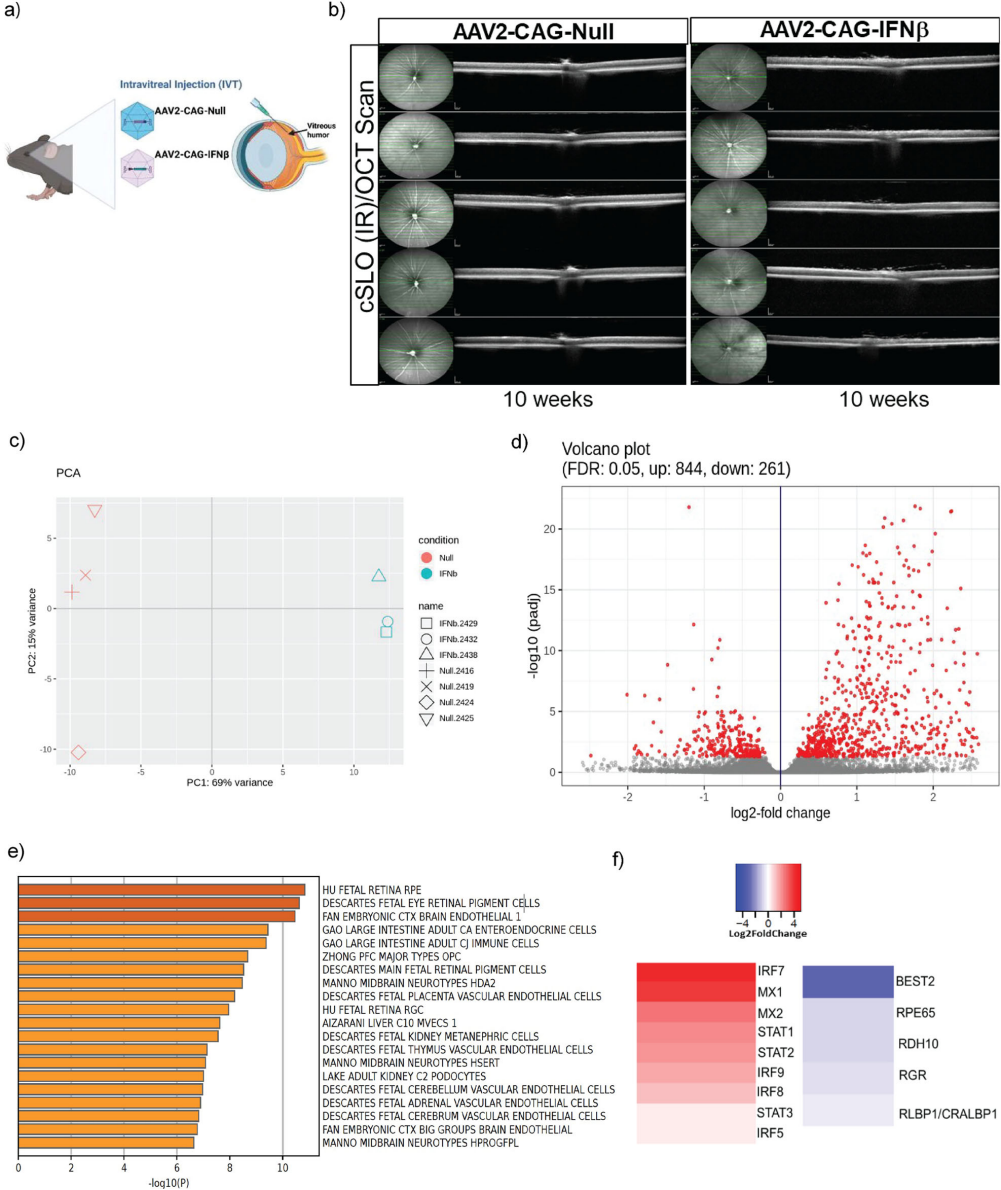
Bulk RNA-seq shows the effects of prolonged IFN-β signaling across RPE and choroid. **a** Schematic showing intravitreal delivery of AAV2-CAG-Null and AAV2-CAG–IFN-β in mouse models. **b** At 10 weeks, there was evidence of inflammation and early evidence of retinal damage and diffuse retinal atrophy was observed in AAV2–CAG–IFN-β-induced mouse eyes using optical coherence tomography (OCT). No retinal damage was observed in AAV2–CAG–Null mouse eyes (*n* = 10 mice/group; 1.2 × 10⁹ GC/μL per eye). **c** Principal component analysis (PCA) revealed a distinct separation between Null-AAV and IFN-β-AAV-treated samples. **d** Volcano plot identified significantly altered genes, including 844 upregulated and 261 downregulated genes (adjusted *p* < 0.05; fold change ≥ 2). **e** Cell-type signature analysis using downregulated genes indicated that RPE cells were the most affected by IFN-β stimulation. **f** Heatmap of upregulated genes showed strong activation of the type I interferon signaling pathway and downregulation of RPE specific *RPE65* and *RDH10* genes.

To further investigate the long-term consequences of sustained IFN-β signaling, we developed an in vivo model using adeno-associated virus (AAV-2) mediated IFN-β overexpression in mouse eyes (Fig. 3a). At 10 weeks post-injection, optical coherence tomography (OCT) revealed early signs of inflammation including hyperreflective foci or appearing in the vitreous and no significant retinal degeneration, including diffuse atrophy and retinal thinning (Fig. 3b). After 10 weeks of IFN-β overexpression, transcriptomic profiling of the RPE/choroidal tissue by bulk RNA sequencing and of the neural retina by scRNA-sequencing was performed. Bulk RNA sequencing of the RPE/choroid revealed widespread activation of type I interferon signaling pathways, as evidenced by the upregulation of canonical interferon-stimulated genes (ISGs), including *STAT1, STAT2, IRF9,* and *MX1*, alongside a concurrent downregulation of genes involved in phototransduction and oxidative metabolism within the RPE/choroid compartment (Fig. 3c–f). Notably, genes associated with visual cycle metabolism, such as *RPE65* and *RDH5*, were significantly suppressed, indicating impaired visual cycle function and potential deficits in chromophore recycling after chronic IFN-β-exposure.

**Fig. 4:**
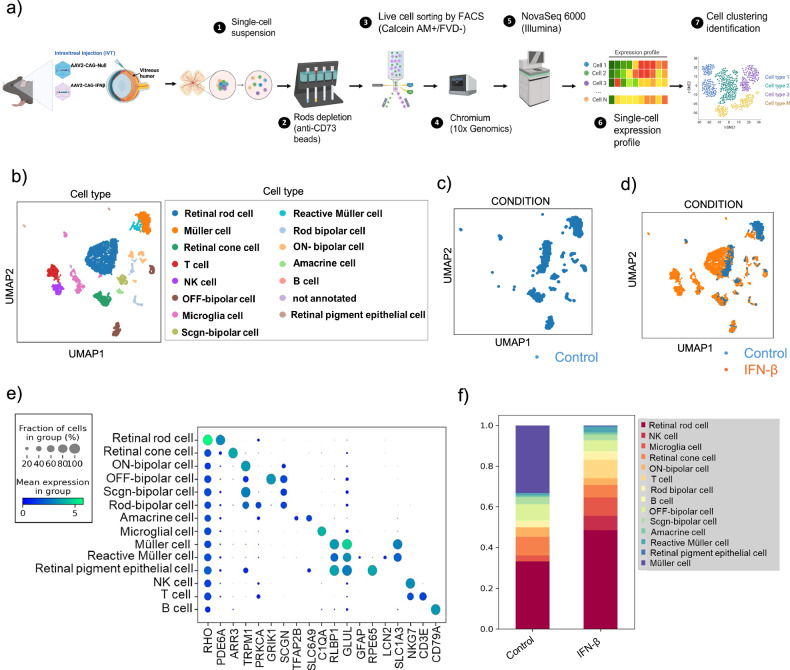
Single cell RNA sequencing shows the effects of prolonged IFN-β signaling across various retinal cell types. **a** A schematic depicts the scRNA-seq analysis pipeline. UMAP visualization of scRNA-seq data from combined IFN-β and Null-AAV treated retinas. **b** Cell clusters colored by treatment conditions; **c** control **d** IFN-β treatment enlarged the populations of T cells, NK cells and microglia cell clusters. **e** Dot plots showing the expression patterns of known marker genes across various retinal cell types. Expression levels are shown as natural logarithm of cp10k (counts per 10k counts) values. **f** Bar chart illustrates the distribution of cell populations in IFN-β-AAV and Null-AAV-treated mouse retinas, with cells colored by type.

To delineate the cellular specificity of transcriptional alterations in the neural retina, we performed single-cell RNA sequencing (scRNA-seq) following 10 weeks of AAV2-mediated IFN-β overexpression. Gene set enrichment analysis (GSEA) revealed alterations of several biological pathways, including visual cycle pathway, along with broad activation of interferon signaling, specifically IFN-β, across multiple retinal cell populations (Fig. S2a, b), including photoreceptors, Müller glia, and retinal ganglion cells, reflecting a global inflammatory milieu (Fig. 4a–f).

**Fig. 5:**
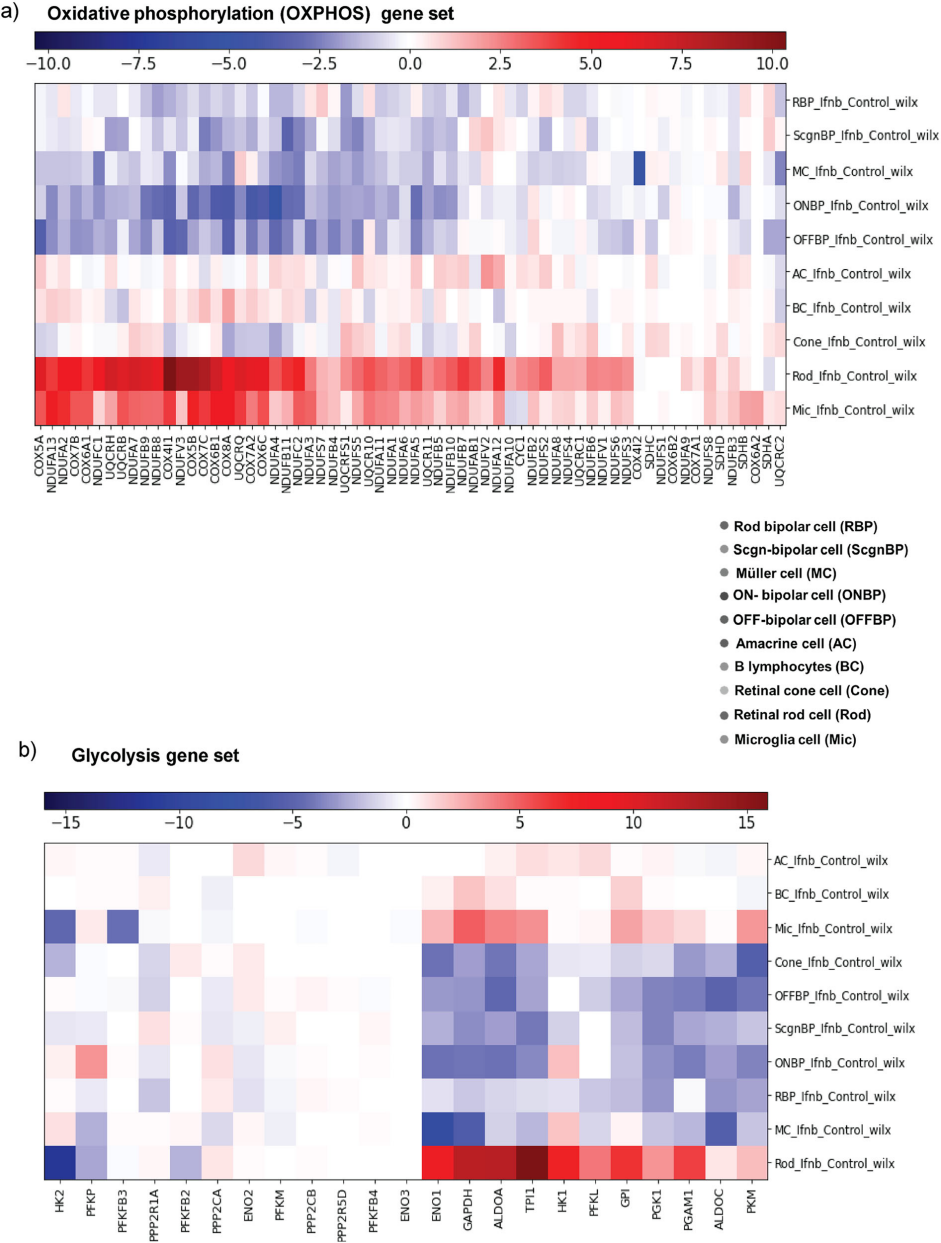
Gene networks in heat map demonstrated altered rod energy metabolism. **a** Heatmap identifies differentially regulated oxidative phosphorylation (OXPHOS) genes in various retinal cells. Oxidative phosphorylation genes like *NDUFA5, NDUFA9, COX4I1*, and *COX5A* were upregulated in rod photoreceptor cells induced by IFN-β. The elevation of OXPHOS genes implies a shift towards more energy production via mitochondrial respiration, which could be in response to increased energy demands or oxidative stress induced by inflammation. **b** Heatmap shows differentially regulated glycolysis genes in various retinal cells. The rod photoreceptor cells show significant reduction of HK2 and increased HK1 observed in IFN-β induced eyes, indicating alterations in glycolytic metabolism in rods upon IFN-β induction. A reduction in HK2 indicates decline in glycolytic flux and a possible compensatory reliance on mitochondrial OXPHO**S** (as shown in part **a**), due to reduced glycolysis. This metabolic alteration could affect the RPE–photoreceptor metabolic coupling, since photoreceptors normally rely heavily on glycolysis and export lactate to RPE.

Rod photoreceptors displayed marked downregulation of genes critical for visual transduction, such as *RHO, GNAT1*, and *PDE6B* (Fig. S3a–c), consistent with impairment mediated by signals from damaged RPE cells in AMD. Additionally, there was an increase in transcripts encoding key mitochondrial enzymes involved in oxidative phosphorylation, including NADH: ubiquinone oxidoreductase subunit A5 (*NDUFA5*), NADH: ubiquinone oxidoreductase subunit A9 (*NDUFA9*), cytochrome c oxidase subunit 4I1 (*COX4I1*), and cytochrome c oxidase subunit 5 A (*COX5A*), along with a reduction in glycolytic genes such as pyruvate kinase M1/2 (*PKM*) and hexokinase 2 (*HK2*), indicating a metabolic switch [62] (Fig. 5a, b). Similarly, Müller glia exhibited a blunted expression of genes linked to energy homeostasis and neurotrophic support [63]. These findings underscore the widespread metabolic and functional reprogramming induced by sustained IFN-β signaling, implicating chronic type I interferon activation in the disruption of visual processing and cellular energy balance that underlie AMD pathology.

### Chronic 20 week IFN-β overexpression induces GA-like retinal degeneration

**Fig. 6:**
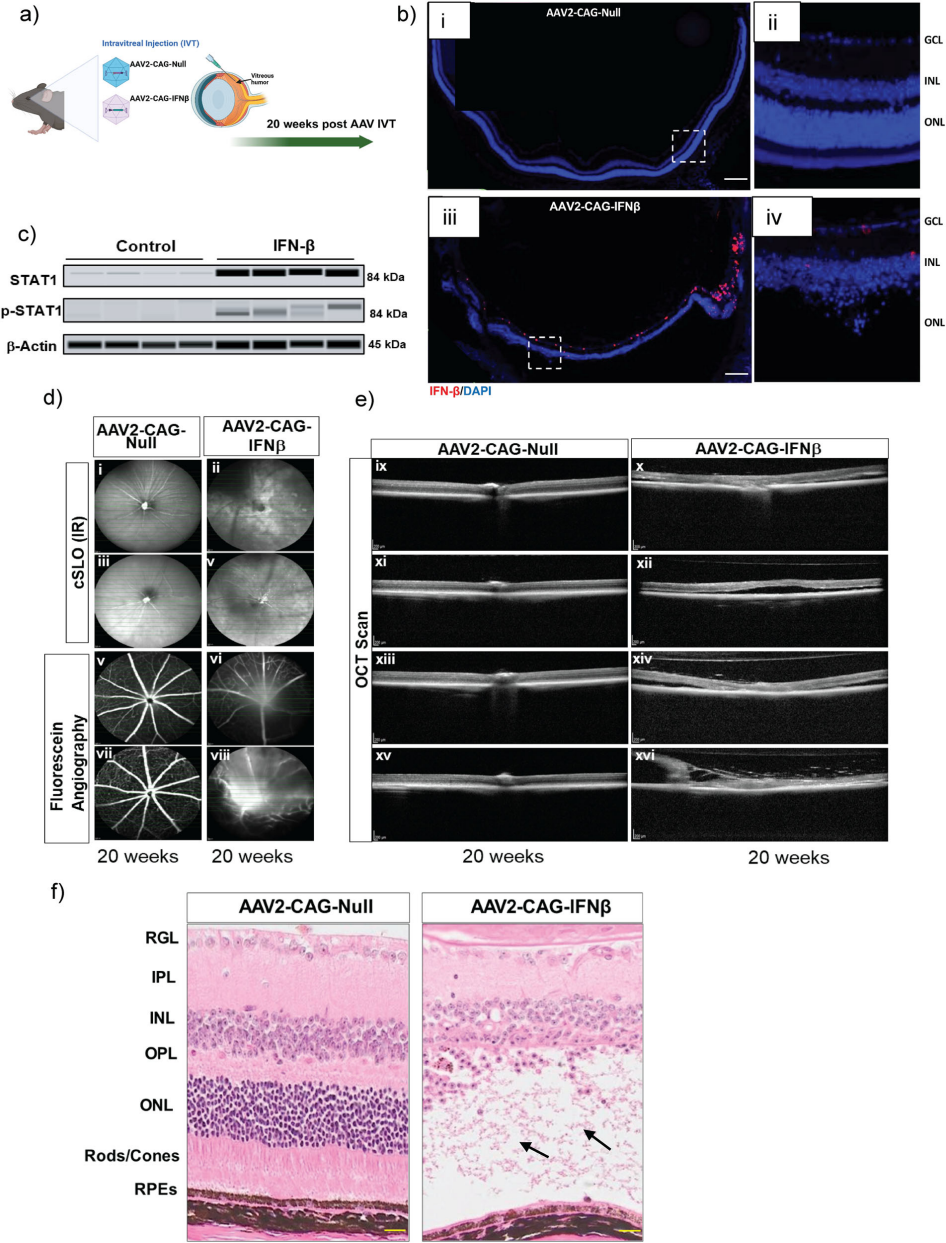
Chronic IFN-β induction (20 weeks) leads to profound retinal degeneration in vivo. **a** Schematic showing intravitreal delivery of AAV2-CAG-Null and AAV2-CAG-IFN-β in mouse models (*n* = 10 mice/group, 1.2 × 10⁹ GC/μL per eye). **b** RNAscope staining of mouse IFN-β (red) demonstrates efficient transduction of retinal cells by AAV2-CAG-IFN-β (panels iii, iv). Scale bar = 100 μm (i, iii) and Scale bar = 50 μm (ii, iv). **c** Western blot analysis confirms activation of the canonical type I IFN pathway in eyes injected with AAV2-CAG-IFN-β at 20 weeks post-injection (*n* = 4 per condition). In vivo imaging of AAV2-CAG-IFN-β-injected eyes shows marked retinal atrophy and degeneration using (**d**) Confocal scanning laser ophthalmoscopy (cSLO), fluorescein angiography, and (**e**) Optical coherence tomography (OCT). Images on the right show subretinal fluid (x, xii, xiv, xvi), and red arrows indicate atrophy and detachment (xvi). **f** H&E staining reveals degeneration of the photoreceptor layer in AAV2-CAG-IFN-β-injected eyes (arrows) compared to AAV2-CAG-Null controls (*n* = 5 per group). Scale bar = 50 μm.

Using our AAV-2 in vivo mouse model of IFN-β overexpression, we observed that chronic AAV2-CAG-IFN-β delivery for 20 weeks sustained IFN-β expression in retinal tissue compared to wildtype controls (Fig. 6a, b). Western blot analysis confirmed JAK/STAT pathway activation, evidenced by increased total and phosphorylated STAT1 in IFN-β-expressing RPE (Fig. 6c).

In vivo non-invasive imaging techniques including confocal scanning laser ophthalmoscopy (cSLO), Optical coherence tomography (OCT) and fluorescein angiography (FA) were used to visualize effects of unperturbed retina after 20 weeks of IFN-β overexpression (Fig. 6d). cSLO revealed areas of widespread RPE atrophy and retinal degeneration in IFN-β exposed mice consistent with GA-like pathology. Similarly, FA showed significant vascular regression and leakage in the same eyes. Optical coherence tomography (OCT) confirmed retinal degeneration and revealed significant disorganization of retinal inner layers (DRIL), an increasingly observed feature in AMD patients (Fig. 6e). These in vivo findings were confirmed by histological analysis which revealed significant photoreceptor and outer nuclear layer degeneration along with RPE abnormalities including RPE vacuolization, which are key features in AMD pathogenesis (Figs. 6f and S4).

### Chronic 20 week IFN-β exposure induces cellular senescence

Loss of RPE-specific markers such as RPE65 and Bestrophin 1 (BEST1) (Fig. S5a), loss of epithelial integrity (Fig. S5b), upregulation of CDKN2A/p16^INK4a^, and increased levels of pro-inflammatory cytokines (Fig. S5d) were observed upon chronic IFN-β exposure in human RPE cells (Fig. S5a–d).

**Fig. 7:**
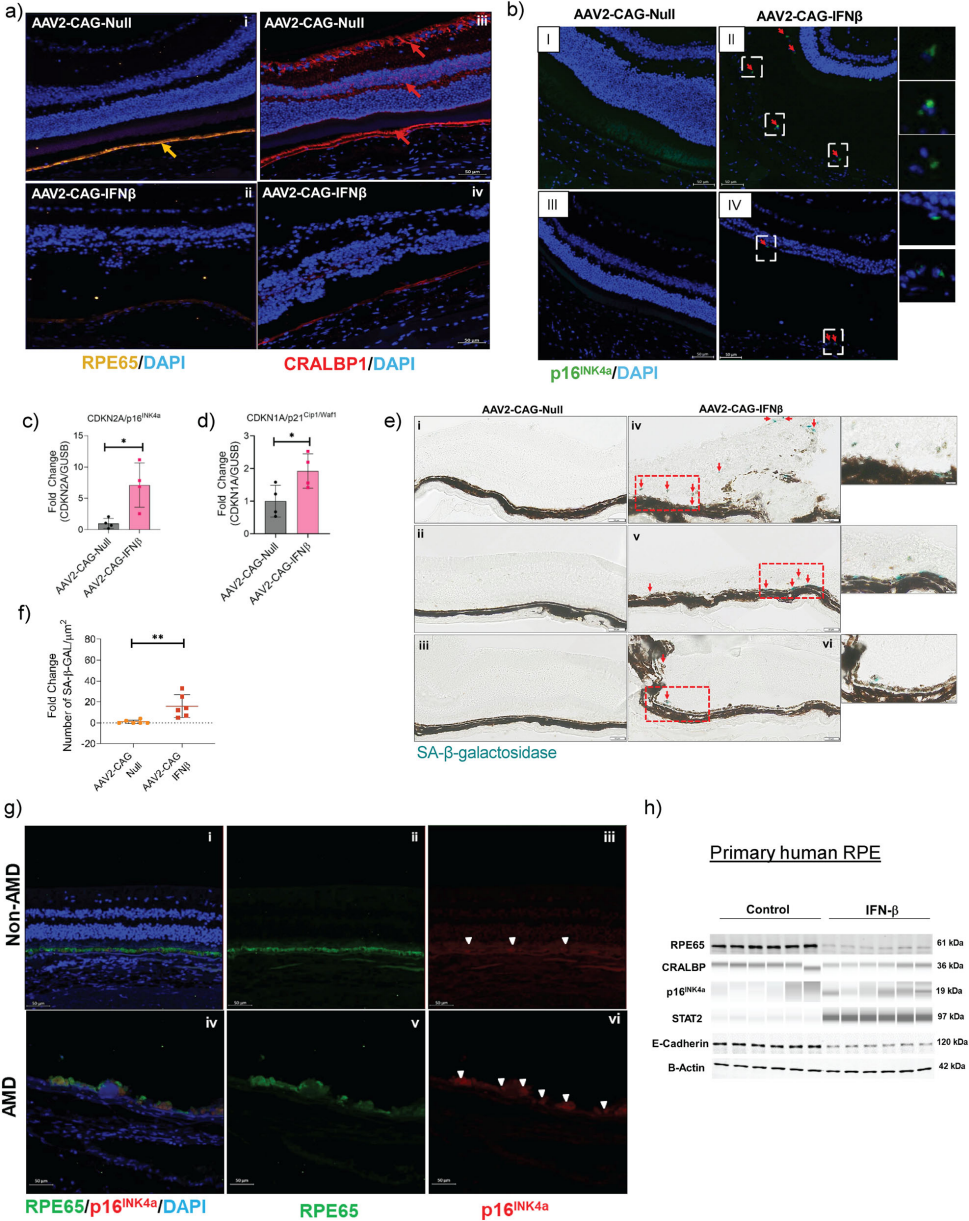
Chronic IFN-β exposure diminishes RPE-specific markers and induces cellular senescence. **a** The expression of the RPE-specific marker RPE65 (orange arrow) was absent in the RPE layer of AAV2-CAG-IFN-β-injected mouse eyes (panel ii) compared to AAV2-CAG-Null controls (panel i). CRALBP1 (cellular retinaldehyde binding protein 1) expression (marked by red arrows) was reduced in the RPE and absent in the neurosensory retina in AAV2-CAG-IFN-β-treated eyes (panel iv) at 20 weeks post-injection. **b** The cell cycle inhibitor protein p16^INK4a^ (green) was upregulated in AAV2-CAG-IFN-β-treated eyes (panels ii, iv, zoomed insets showing p16^INK4a^ positive cells), but not in AAV2-CAG-Null controls (panels i, iii) at 20 weeks post-injection (*n* = 4). Scale bar = 50 μm (zoomed insets, Scale bar = 20 μm). Senescence marker genes (**c**) *CDKN2A/p16*^*INK4a*^ and (**d**) *CDKN1A/p21*^*Cip1/Waf1*^ were significantly upregulated in RPE/choroid tissues 10 weeks after IFN-β AAV injection (*n* = 4, *p* < 0.05). **e** Senescence-associated (SA)-β-galactosidase staining of cryosections demonstrated increased β-gal-positive cells in AAV2-CAG-IFN-β-injected eyes (panels iv–vi) compared to AAV2-CAG-Null controls (panels i–iii). Scale bar=50 μm. **f** Quantification of SA-β-gal-positive cells using the HALO® system (Indica Labs v3.0.311.261) revealed a significant increase in the IFN-β group relative to controls (*n* = 6). **g** Immunofluorescence staining of human non-AMD donor eye tissues demonstrated co-localization of the RPE marker RPE65 (panels i, ii) with the cell cycle inhibitor p16^INK4a^ (panels i, iii). A reduction of RPE65 expression was observed in AMD donor tissues (panel v) compared to non-AMD controls (panel ii). RPE cells showed increased p16^INK4a^ staining in AMD eyes (panels iv, vi) (*n* = 4). Scale bar=50 μm. **h** Western blot analysis showed increased senescence markers and decreased E- cadherin, indicating reduced RPE epithelial integrity in primary human RPE cells stimulated with IFN-β for 7 days. Values represent mean ± s.d. from triplicate experiments (*n* = 3). Statistical significance was determined using an unpaired *t*-test. *p < 0.05, **p < 0.01.

Similarly, the expression of the RPE-specific marker RPE65 was absent in the RPE layer of AAV2-CAG-IFN-β-injected mouse eyes (Fig. 7a, panel ii) compared to AAV2-CAG-Null controls (Fig. 7a, panel i). CRALBP1 expression was reduced in the RPE and absent in the neurosensory retina in AAV2-CAG-IFN-β-treated eyes (Fig. 7a, panel iv) at 20 weeks post-injection. The cell cycle inhibitor protein p16^INK4a^ was upregulated in AAV2-CAG-IFN-β-treated eyes (Fig. 7b, panels ii, iv), but not in AAV2-CAG-Null controls (Fig. 7b, panels i, iii) at 20 weeks post-injection. Senescence marker genes *CDKN2A/p16*^*INK4a*^ (Fig. 7c) and *CDKN1A/p21*^*Cip1/Waf1*^ (Fig. 7d) were significantly upregulated in RPE/choroid tissues 10 weeks after IFN-β AAV injection.

Senescence-associated (SA)-β-galactosidase staining of cryosections demonstrated increased β-gal-positive cells in AAV2-CAG-IFN-β-injected eyes (Fig. 7e, panels iv–vi) compared to AAV2-CAG-Null controls (Fig. 7e, panels i–iii). Quantification of SA-β-gal-positive cells revealed a significant increase in the IFN-β group relative to controls (Fig. 7f). Immunofluorescence staining of human non-AMD donor eye tissues demonstrated co-localization of the RPE marker RPE65 (Fig. 7g, panels i, ii) with the cell cycle inhibitor p16^INK4a^ (Fig. 7g, panels i, iii). A reduction of RPE65 expression was observed in AMD donor tissues (Fig. 7g, panel v) compared to non-AMD controls (Fig. 7g, panel ii). RPE cells showed increased p16^INK4a^ staining in AMD eyes (Fig. 7g, panels iv, vi). Western blot analysis showed increased senescence markers and decreased E-cadherin, indicating reduced RPE epithelial integrity in primary human RPE cells stimulated with IFN-β for 7 days (Fig. 7h).

### Therapeutic modulation of STING signaling

Our findings collectively define STING as a central driver of chronic retinal inflammation through the coordinated upregulation of IFN-β and IL-17A, two key pro-inflammatory cytokines that act synergistically to disrupt retinal homeostasis. The convergence of STING signaling on both the interferon and interleukin pathways underscores its pivotal role in sustaining immune activation within the retina. Importantly, these results identify STING not only as a molecular switch that initiates type I interferon and IL-17A signaling, but also as a potent amplifier of interconnected cytokine networks that promote progressive retinal degeneration.

To evaluate the translational potential of STING inhibition, we used the selective and reversible small molecule STING inhibitor, SN-011 [64]. Reversible inhibition with SN-011 offers significant advantages over irreversible STING inhibitors by allowing precise control over inflammatory signaling suppression while maintaining essential immune functions like anti-viral immune response. This controlled modulation minimizes toxicity in RPE cells and enables flexible dosing strategies, including treatment cycling, characteristics particularly valuable for managing progressive, chronic conditions like AMD.

**Fig. 8:**
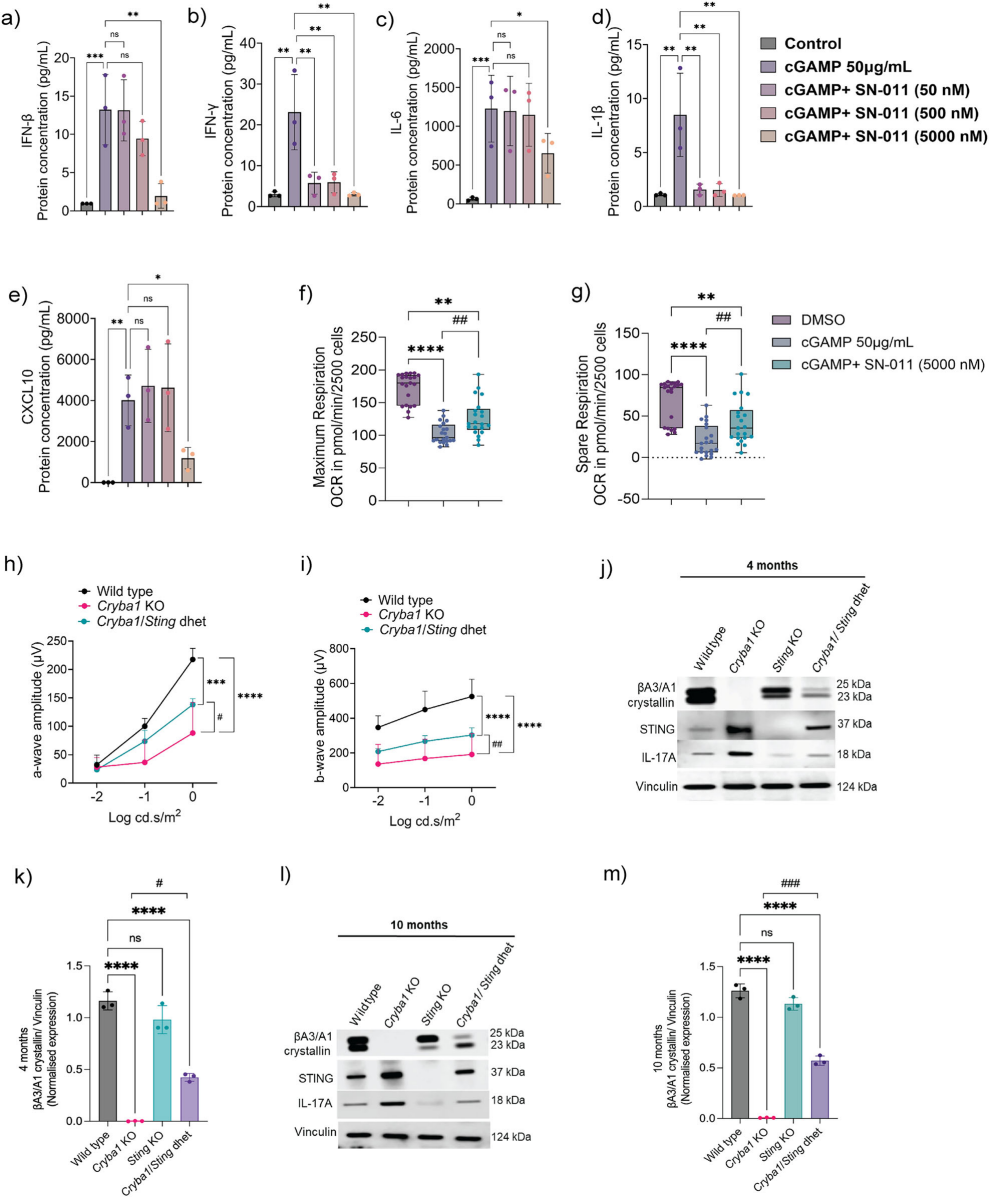
Therapeutic modulation of the STING–IFN-β-IL-17A inflammatory axis. **a** Mature human RPE (hRPE) cells were treated with 50 μg/mL cGAMP and varying concentrations of the STING inhibitor SN-011 for 24 h. Cell supernatants were collected, and levels of (**a**) IFN-β, (**b**) IFN-γ, (**c**) IL-6, (**d**) IL-1β and (**e**) CXCL10 were quantified using a Luminex assay. Values represent mean ± s.d, *n* = 3 independent experiments. Box plots show data points for (**f**) maximum mitochondrial respiration and (**g**) spare respiratory capacity, measured by Seahorse XF assays. *n* = 7 biological replicates in triplicate. Values represent mean ± s.d from biological replicates. Statistical significance was assessed using one-way ANOVA with the Friedman test. *p < 0.05, **p < 0.01, ***p < 0.001, and ****p < 0.0001 indicate statistical significance between cGAMP and (DMSO) and or SN-011 treatments. ^##^*p* < 0.01 indicates statistical significance between cGAMP alone and cGAMP combined with SN-011 treatment conditions. ns =not significant. Electroretinography (ERG) recordings of 24-h dark-adapted wild type, *Cryba1* KO and *Cryba1/Sting* dhet mice showed decline in scotopic (**h**) a-wave and (**i**) b-wave responses at three light intensities (0.01, 0.1, and 1 cd s/m²) in *Cryba1* KO mice, compared to wild type indicating retinal function impairment. *Cryba1/Sting* dhet mice showed noticeable rescue in both (**h**) a- and (**i**) b-waves responses, compared to *Cryba1* KO mice. # indicates statistical significance between *Cryba1* KO and C*ryba1/Sting* dhet mice (*n* = 3). **j** Western blot and (**k**) densitometry analysis of RPE lysates from 4-month-old mice showing rescue of protein expression levels of βA3/A1-crystallin, STING, and IL-17A in *Cryba1/Sting* dhet mice relative to *Cryba1* KO mice. WT and *Sting* KO mice were used as controls (*n* = 3). **l** Western blot and **m** densitometric quantification of RPE lysates of 10-month-old WT, *Cryba1* KO, *Sting* KO, and *Cryba1/Sting* dhet mice showed similar and notable rescue of the βA3/A1-crystallin, STING, and IL-17A protein levels in dhet mice, relative to *Cryba1* KO (*n* = 3). Values represent the mean ± s.d (*n* = 3). Statistical analysis was performed using two-way ANOVA with Dunnett’s multiple comparisons test., ***p < 0.001, ****p < 0.0001, ^####^*p* < 0.0001.

Pharmacological blockade of STING significantly reduced cGAMP-induced expression of inflammatory cytokines, including IFN-β and IFN-γ (Fig. 8a, b), along with IL-6, IL-1β and CXCL10 (Fig. 8c–e) as well as IL-10, IL-12p70, IFN-α, IFN-λ2, IFN-λ3, TNF-α and GM-CSF (Fig. S5e–k) in human RPE cells. The concurrent suppression of both type I and type II interferons suggests that STING activation lies upstream of pro-inflammatory signaling in RPE cells. Notably, SN-011 treatment also restored mitochondrial function in primary human RPE cells (Fig. 8f, g). Seahorse metabolic analysis revealed improved maximal respiration and spare respiratory capacity following STING inhibition (Fig. 8f, g), highlighting its therapeutic potential in alleviating mitochondrial dysfunction and inflammation in AMD.

Functional rescue was demonstrated through electroretinography (ERG), where *Cryba1*/*Sting* dhet mice exhibited significantly increased a- and b-wave amplitudes compared to *Cryba1* KO mice (Fig. 8h, i), indicating partially preserved rod function and retinal integrity. Our longitudinal analysis at 4- and 12-month time points reveals the dual role of STING in AMD pathogenesis and highlights the importance of early intervention. At the early stage (4 months), protein analysis from RPE lysates of *Cryba1/Sting* dhet mice showed partial preservation of βA3/A1-crystallin and reduced STING levels, accompanied by decreased IL-17A expression, compared to age-matched *Cryba1* KO mice (Fig. 8j, k). These molecular changes were associated with preserved retinal function, as evidenced by improved ERG responses (Fig. 8h, i). Importantly, this protective effect persisted at 10 months of age, where *Cryba1/Sting* dhet mice continued to exhibit lower IL-17A levels and maintained partial expression of βA3/A1-crystallin and STING in RPE lysates relative to age-matched *Cryba1* KO mice (Fig. 8l, m). These findings indicate that partial suppression of STING signaling in the RPE at an early age not only attenuates chronic inflammation but also preserves retinal function. Thus, targeting the STING pathway during the early stages of disease may help prevent the progressive retinal degeneration characteristic of AMD.

**Fig. 9:**
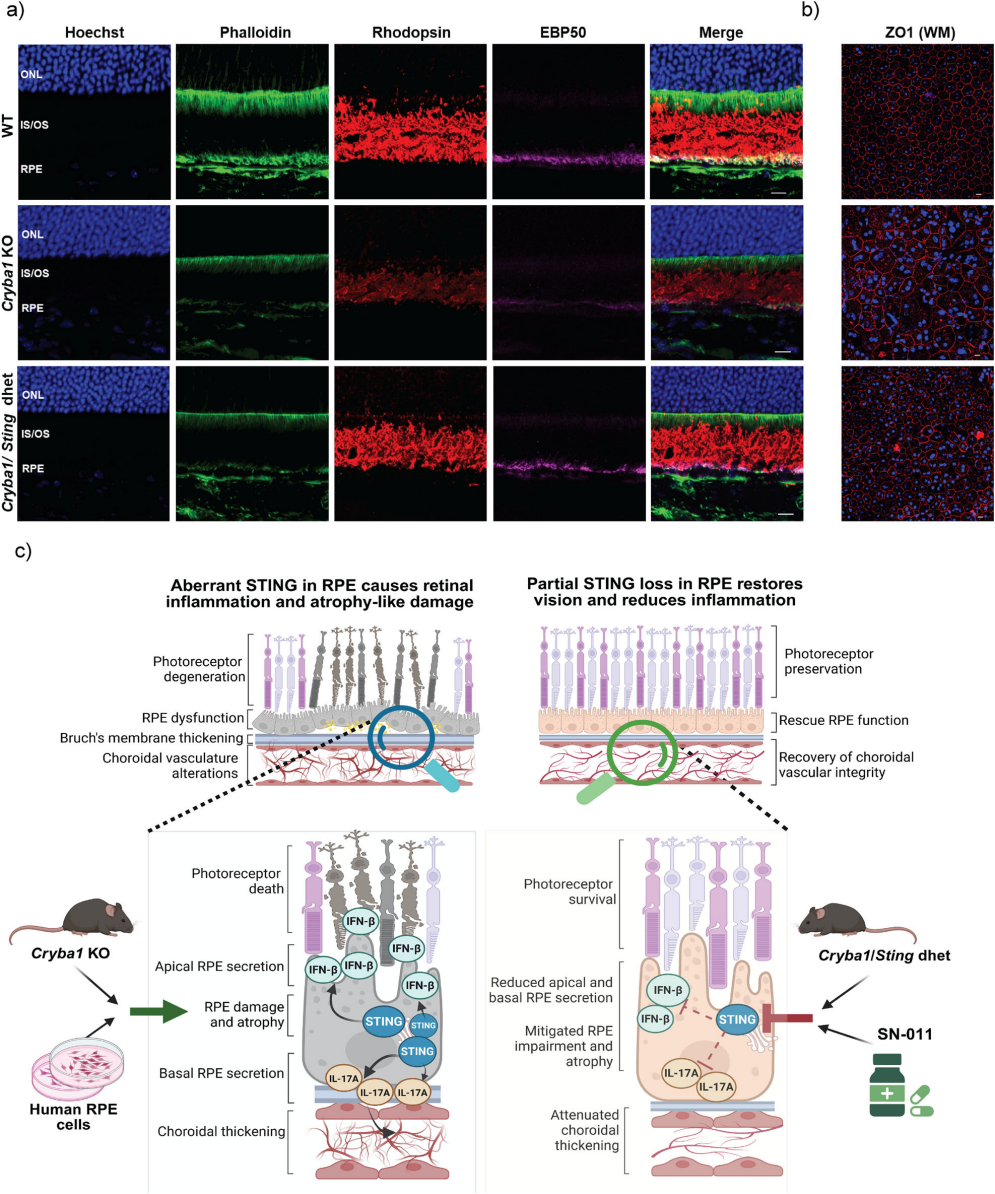
Rescue of retinal structure and function in *Cryba1/Sting* dhet mice. **a** Immunohistochemical analysis of retinal sections from 10-month-old mice demonstrates disrupted apical villi of the retinal pigment epithelium (RPE) in *Cryba1* KO mice, as shown by decreased and disorganized Phalloidin (green) and EBP50 (magenta) staining compared to age-matched wild-type controls. Additionally, a marked reduction in rhodopsin expression (red) is observed in *Cryba1* KO retinas. Both structural abnormalities and rhodopsin expression are partially restored in the *Cryba1/Sting* dhet retinas, indicating a protective effect of partial *Sting* deletion and *Cryba1* upregulation (*n* = 3). Scale bar = 10 μm. **b** Immunostaining of RPE flat mounts with ZO-1 highlights tight junction integrity and morphology. In wild-type mice, ZO-1 staining (red) reveals a regular cobblestone pattern, which is disrupted in *Cryba1* KO mice, indicating loss of junctional organization and epithelial structure. This junctional integrity is substantially restored in the *Cryba1/Sting* dhet group, suggesting rescue of RPE architecture (*n* = 3). **c** The schematic highlights the central role of STING-mediated inflammation in retinal degeneration like AMD, as evident from our studies using mouse models and human iPSC-derived RPE cells as tools. Our findings suggest that targeted STING inhibition could be a promising therapeutic strategy to preserve retinal structure and function in AMD.

The significance of this early rescue becomes apparent when examining aged mice at 10 months, where comprehensive molecular rescue was sustained. While *Cryba1* KO mice showed progressive deterioration with noticeable decreases in EBP50 (magenta), phalloidin-labeled apical microvilli (green), and rhodopsin (red) compared to wildtype controls, these degenerative changes were ameliorated in *Cryba1*/*Sting* dhet mice (Figs. 9a and S5l–n). Similarly, RPE integrity was robustly maintained over time, as evidenced by ZO-1 immunostaining (red) on RPE flatmounts, where 10-month-old *Cryba1* KO mice displayed severely abnormal cobblestone-like RPE morphology while the *Cryba1/Sting* dhet group retained normal RPE architecture and ZO-1 expression (Fig. 9b).

Our study demonstrates that strategically timed inhibition of the cGAS/STING pathway rescues both morphological RPE changes and functional integrity, with early intervention providing compounding benefits that persist with age, making our temporal data highly clinically relevant for understanding optimal therapeutic windows in AMD treatment (Fig. 9c).

## Discussion

Our investigation establishes STING as a master regulator of chronic retinal inflammation and degeneration in AMD, revealing a previously unrecognized mechanism that coordinates both photoreceptor death and choroidal dysfunction through spatially organized cytokine secretion. The identification of enriched IFN-β secretion from the apical surface and IL-17A secretion from the basal surface of polarized AMD patient-derived RPE cells provides the first mechanistic explanation for the selective vulnerability of photoreceptors and concurrent choroidal abnormalities that characterize GA [65, 66]. The stage-dependent redistribution of STING from apical to cytoplasmic localization in human AMD eyes, coupled with parallel IFN-β activation, demonstrates that STING undergoes a fundamental functional change during disease progression [67]. While apical STING localization in healthy RPE likely supports normal immune surveillance, the diffuse cytoplasmic distribution observed in AMD suggests dysregulated activation that drives pathogenic inflammation [68]. This spatial reorganization parallels disease severity, with maximal STING expression in end-stage disciform scars [39], establishing this pathway as a consistent feature of AMD pathology. These findings are particularly significant considering recent work demonstrating that age-associated mitochondrial DNA release and cGAS/STING activation represent fundamental aging mechanisms across multiple organs [13] and species [69, 70]. Our results extend these observations by showing that in AMD, this age-related inflammatory pathway becomes pathologically amplified and spatially reorganized within the RPE.

The polarized cytokine secretion pattern reveals significant precision in AMD pathogenesis. Selective apical IFN-β secretion by AMD patient-derived iPSC-RPE cells provides a direct pathway for photoreceptor toxicity, explaining why these neurons are preferentially vulnerable [71, 72]. Simultaneously, enriched basal IL-17A secretion creates a mechanism for choroidal dysfunction [73] that operates independently of photoreceptor damage [74]. This spatial organization suggests that AMD pathology results from a precisely coordinated attack on multiple retinal compartments rather than random inflammatory damage. The feed-forward inflammatory loop between IFN-β and IL-17A represents a crucial mechanistic insight explaining AMD’s progressive nature. STING activation simultaneously triggers both arms of this loop: IFN-β secretion directly damages photoreceptors while amplifying IL-17A production, which compromises choroidal vasculature and further activates inflammatory cascades. This self-perpetuating cycle provides a mechanistic basis for understanding why AMD accelerates over time and why anti-inflammatory therapies targeting individual mediators have been largely unsuccessful [75]. The involvement of both type I and type II interferons in this loop suggests that multiple interferon pathways converge to amplify IL-17A expression and sustain chronic inflammation [76].

Our single-cell RNA sequencing analysis following chronic IFN-β exposure reveals that sustained interferon signaling fundamentally reprograms the entire retinal cellular landscape. Broad activation of interferon-stimulated genes across all major retinal cell types—photoreceptors, Müller glia, and retinal ganglion cells—demonstrates that STING-mediated inflammation extends far beyond the RPE to create a global inflammatory environment. The selective downregulation of visual transduction genes in rod photoreceptors, including *RHO*, *GNAT1*, and *PDE6B*, provides molecular evidence for functional visual impairment in AMD patients. Concurrent metabolic reprogramming from glycolysis to oxidative phosphorylation indicates that chronic interferon exposure forces photoreceptors into an energetically unfavorable state that compromises light responsiveness [77]. The onset of cellular senescence marks a pivotal transition where reversible cellular dysfunction progresses into irreversible tissue degeneration [78]. Our demonstration that chronic IFN-β exposure rapidly induces senescence markers, including p16^INK4a^ and p21^Cip1/Waf1^, while eliminating RPE-specific markers like RPE65 and CRALBP1, establishes a direct mechanistic link between STING activation and loss of RPE identity in advanced AMD. Senescent cells not only represent functional loss but also create additional inflammatory sources through the senescence-associated secretory phenotype [79], potentially expanding dysfunction beyond initial STING activation sites.

The rescue of AMD-like phenotypes through both pharmacological STING inhibition and partial genetic ablation demonstrates this pathway’s therapeutic potential. The effectiveness of reversible STING inhibitor SN-011 [64] in suppressing multiple inflammatory cytokines while restoring mitochondrial function suggests that STING inhibition can address both inflammatory and metabolic dysfunction characteristic of AMD. The restoration of mitochondrial respiration and spare respiratory capacity is particularly significant given the retina’s high metabolic demands and established role of mitochondrial dysfunction in AMD pathogenesis [80]. The preservation of retinal function demonstrated by improved electroretinographic responses in rescued mice indicates that STING inhibition maintains physiological retinal capabilities rather than simply preventing morphological damage. The complementary mechanism between CRYBA1 and STING provides insight into how lysosomal dysfunction [49, 53, 81] and inflammatory signaling [52, 82, 83] interact in AMD pathogenesis. STING upregulation following CRYBA1 loss suggests that lysosomal impairment creates conditions favoring STING activation [84], possibly through accumulation of cytosolic DNA or damage-associated molecular patterns [85]. The rescue in heterozygous mice indicates that maintaining partial CRYBA1 function while reducing STING activity creates a balanced cellular environment, preventing pathological progression. This lysosome-STING connection is particularly relevant given recent evidence that lysosomal dysfunction [86] and impaired mitophagy [86] contribute to age-related mitochondrial DNA accumulation and subsequent cGAS/STING activation. Our study focuses on the *Cryba1* KO model as a proof-of-concept system. To fully establish the clinical relevance of STING-mediated pathways in AMD, validation across complementary disease models like *Akt2* KI [87] and *Cfh* transgenic mouse models [88, 89] will be necessary to demonstrate mechanistic convergence beyond this single experimental context.

Our findings establish STING as the first identified master regulator capable of simultaneously controlling multiple pathogenic processes in AMD. Unlike previous therapeutic targets addressing individual disease aspects, STING inhibition disrupts fundamental mechanisms driving both photoreceptor death and choroidal dysfunction while maintaining essential immune surveillance functions. The stage-dependent nature of STING activation suggests therapeutic intervention would be most effective during early disease progression before irreversible senescence becomes widespread. Importantly, our therapeutic approach differs fundamentally from direct STING inhibition strategies that might compromise immune function. Recent studies have shown that while STING inhibitors can reduce age-associated neuroinflammation [90, 91], long-term systemic inhibition raises concerns about pathogen susceptibility [22, 92]. Our demonstration that targeting upstream triggers of STING activation (through lysosomal function restoration or mitochondrial quality control) can achieve therapeutic benefits while preserving essential innate immune responses represents a more clinically viable approach. The demonstration that reversible STING inhibition can restore normal cellular function while preventing pathological inflammation provides a clear pathway for clinical translation. Given the established safety profile of STING pathway modulators in other disease contexts [93, 94] and the urgent need for effective AMD therapies [5], our findings support the rapid advancement of STING-targeted interventions to clinical trials. The ability to monitor STING activation and localization as biomarkers of disease progression [95] and therapeutic response [95] further enhances the translational potential of this approach.

Our work contributes to the growing understanding that aging-related diseases represent not merely the accumulation of random damage, but rather the pathological amplification of conserved aging mechanisms [96]. The identification of STING as a central hub integrating lysosomal dysfunction, mitochondrial damage, and inflammatory signaling in AMD provides a framework for understanding how multiple age-related stressors converge to drive tissue degeneration. This mechanistic convergence may explain why AMD incidence increases so dramatically with age [5] and why interventions targeting individual pathways have shown limited success [75, 97]. Furthermore, our findings suggest that other age-related retinal diseases may share similar STING-mediated pathogenic mechanisms, opening new therapeutic avenues for diabetic retinopathy, glaucoma, and inherited retinal degenerations where inflammation and metabolic dysfunction play central roles. The development of STING-targeted therapies for AMD could therefore have broader applications across the spectrum of retinal diseases.

## Materials and methods

### Animals

RPE-specific βA3/A1-crystallin conditional knockout (*Cryba1* cKO) [49, 52, 53], as well as global *Cryba1* KO [53, 98] mice were generated on a C57BL/6J background as previously described. All animal studies were conducted in accordance with the Guide for the Care and Use of Animals (National Academy Press) and were approved by the Johns Hopkins University Animal Care and Use Committee. Both male and female mice were used in this study. All mice that were used in this study were negative for the RD8 mutation. Animals were euthanized as per approved guidelines, and the RPE was harvested for downstream experiments.

Six- to eight-week-old wild type male C57BL/6J mice were purchased from Charles River Laboratories, France to develop an in vivo model using adeno-associated virus (AAV2)-mediated IFN-β overexpression in mouse eyes. The mice were kept in individually ventilated cages at a temperature of 22 °C, humidity of 50%, and were on a 12-h light/dark cycle with ab libitum access to standard rodent chow and water. All mice underwent 7 days of acclimation before performing the experiment. All studies involving animals were carried out on the animal experimentation license (BS-2676) and were approved by the CVO (Cantonal Veterinary Office) Basel-Stadt and the FSVO (Federal Food Safety and Veterinary Office). All animal care and experimental procedures were in accordance with Swiss legislation and guidelines for animal care and usage. OCT and fluorescein angiography (Spectralis, Heidelberg Engineering, Germany) were performed to evaluate retinopathy and retinal degeneration. Mice were anaesthetized with a cocktail of fentanyl 0.05 mg/kg, medetomidine 0.5 mg/kg, and midazolam 5 mg/kg by subcutaneous injection. Pupils were dilated with tropicamide (0.5%) (Théa Pharma, Belgium). Fluorescein angiography scans were taken after subcutaneous injection of sodium fluorescein (2% wt/vol.). Anesthesia was reversed with a subcutaneous injection of naloxone 1.2 mg/kg, atipamezole 2.5 mg/kg, and flumazenil 2.5 mg/kg.

Generation of *Il17a* KI mice: The *Il17a* KI mice were generated by Cyagen Biosiences (Santa Clara, CA) as a paid service. Briefly, the gRNA to mouse ROSA26 gene [99], the donor vector containing CAG Promoter-loxP-6*SV40 pA-loxP-Kozak-Mouse Il17a CDS-IRES-tdTomato-rBG pA cassette, and Cas9 mRNA were co-injected into fertilized mouse eggs to generate targeted conditional KI offspring. F0 founder animals, identified by PCR followed by sequence analysis, were bred to wildtype mice to test germline transmission and F1 animal generation (heterozygotes). These heterozygotes were intercrossed to generate homozygous targeted mice, which were then bred with *Best1*-Cre for RPE-specific KI of IL17a.

Generation of *Cryba1*/*Sting* double het (dhet) mice: *Cryba1* KO homozygous mice were bred with *Sting* KO [53] (The Jackson Laboratories, USA, stock #036638) to generate the F1 generation of heterozygote double KO mice.

### AAV-2 vectors expressing IFN-β and intravitreal injection (IVT) administration of AAVs

The AAV-2 vectors were produced by Vector Biolabs (Malvern, PA, USA) at a concentration of 8.7 × 10^12^ GC/mL (AAV2-CAG-m-IFN-β−WPRE) and 3.5 × 10^12^ GC/mL (AAV2-CAG-Null). The AAV2-CAG-m-IFN-β-WPRE vector utilized the cytomegalovirus early enhancer/chick β actin (CAG) promoter to drive the expression and synthesize mouse IFN-β peptides, and the woodchuck hepatitis virus posttranscriptional regulatory element (WPRE) allows for a sufficient level of expression with integrated vectors. The AAV2-CAG-Null contained the same promoter but without the shuttle DNA sequence. AAV vector stocks were thawed from the storage at -80 °C on the day of injection. Vectors were diluted to a titer of 1.2 × 10^9^ GC/μL in 1x phosphate-buffered saline (PBS) and placed on ice until the injection. Mice were induced with isoflurane (ISO) in an induction chamber and maintained under anesthesia via nose-cone delivery. The body temperature was maintained by using a heating pad until animals were fully recovered from anesthesia. Prior to IVT injection, topical administration of tropicamide (0.5%) (Théa Pharma, Belgium) was done for pupil dilation. The anesthetized mouse was placed under a surgical microscope, and a dorsal sclerotomy was made with a 33-gauge needle approximately 1.5 mm below the limbus. A 33-gauge blunt-tipped needle connected to a 100μL Hamilton syringe was attached to a MicroSyringe Pump Controller (Micro4, World Precision Instruments, Inc., USA). The blunt-tipped needle was then inserted into the vitreous space through the sclerotomy, and 1 μL of the vector suspension was injected. The same procedure was performed on the opposite eye. After IVT injection, antibiotic ointment was applied to both eyes. Mice were returned to the cage on a heating pad until they were fully recovered from the anesthesia. A drop of lubricating eye drops (Genteal Gel, Alcon Laboratories, USA) was applied to the cornea to prevent drying and damage during recovery.

### Eye tissue preparation

Mouse eyes were enucleated post euthanasia and collected into 1.5 mL tubes, snap-frozen on dry ice, and stored at -80 °C. To obtain total eye lysate, the whole eye was placed into a 0.5 mL Precellys tube (Bertin Technologies, France) with 50μL of RIPA lysis buffer plus Halt Protease and Phosphatase Inhibitor Cocktail (Thermo Scientific, USA). Eye lysates were prepared using a Precellys 24 tissue homogenizer (Bertin Technologies, France) by shaking for 15 seconds at 5500 RPM followed by centrifugation at 12000 rpm for 3 min at 4 °C. The protein concentration of each lysate was determined by Direct Detect® Spectrometer (Millipore, USA).

### RNA sequencing of RPE/choroid tissue

Total RNA was extracted from 8 mouse RPE/choroid tissues using a RNeasy Mini Kit (Qiagen, USA, Cat. #74104) following the manufacturer’s protocol. Isolated RNA was eluted in nuclease free water, and library preparation and NovaSeq Sequencing were performed at Azenta, USA. Samples were quantified using Qubit 4.0 Fluorometer (Life Technologies, USA), and RNA integrity was checked with RNA Kit on Agilent 5300 Fragment Analyzer (Agilent Technologies, USA). RNA sequencing libraries were prepared using the NEBNext Ultra II RNA Library Prep Kit for Illumina following the manufacturer’s instructions (NEB, USA). Briefly, mRNAs were first enriched with Oligo(dT) beads and subsequently fragmented at 94 °C for 15 min. First and second strand cDNAs were then synthesized. The resulting cDNA fragments were end repaired, adenylated at 3’ ends, and ligated to universal adapters, followed by index addition and library enrichment by limited-cycle PCR. Library quality was assessed using the NGS Kit on an Agilent 5300 Fragment Analyzer (Agilent Technologies, USA), and concentrations were determined with a Qubit 4.0 Fluorometer (Invitrogen, USA). The sequencing libraries were multiplexed and loaded on the flowcell on the NovaSeq 6000 (Illumina, USA) according to manufacturer’s protocol. The samples were sequenced using a 2 × 150 Pair-End (PE) configuration v1.5. Image analysis and base calling were conducted by the NovaSeq Control Software v1.7 on the NovaSeq instrument. Raw sequence data (bcl files) generated from the source were converted into fastq files and de-multiplexed using the Illumina bcl2fastq program version 2.20. One mismatch was allowed for index sequence identification.

### Single-cell preparation and sequencing of retinal tissue

Neural Tissue Dissociation Kit-Postnatal Neurons (MACS, Cat. #130-094-802) was used for retinal tissue dissociation. The retinal dissociation protocol was adopted from previous publications with modifications [100]. Retinas (both RPE and neural retina; *n* = 4 for Null-AAV, *n* = 4 for IFN-β-AAV) were dissected out from the anterior segment under normal light in ice-old D-PBS w/ Ca^2+^/Mg^2+^. The retinas were then transferred into pre-warmed buffer containing Buffer Z and Enzyme P, and then incubated for 15 min at 37 °C with gentle shaking using a Thermomixer (Eppendorf, Germany). Next, enzyme mix 2 (Buffer Y plus Enzyme A) was added, and the retinas were dissociated by gently pipetting up and down using a cropped plastic transfer pipette. The tissue mixture was incubated for 10 min at 37 °C on the Thermomixer before adding an additional 7.5 μL Enzyme mix 2. The retinal tissue was further dissociated by trituration, and the suspension was filtered through a 70 μm cell strainer and centrifuged for 10 min at 200 × *g* (4 °C). Dissociated retinal cells were incubated with anti-CD73-PE (MACs, Cat. #130-102-616, 10^7^ cells per tube) followed by anti-PE microBeads (MACs, Cat. #130-048-801, 10^7^ cells per tube) to deplete rods based on a previously published procedure [100]. Calcein violet AM (Invitrogen, USA, Cat. #C34858) and eFluor780 (Invitrogen, USA, Cat. #65-0865) were used to enrich live cells. Incubation was done in the dark at 4 °C for 20 min. CD73 negative cells were selected via MS Columns (MACs, Cat. #130-042-201) through a Mini MACs Separator (MACs, Milltenyi Biotec, Germany). Viable CD73 negative cells were further enriched by using a FACS sorter (Aria Fusion, BD Biosciences, USA) with gating of Calcein AM positive, eFluor780 negative cell populations. Single, viable cell suspensions were diluted to 1000 cells/μL in PBS with 0.04% BSA for loading into 10x Chromium Single Cell v 3.1 Chips. Following collection, cDNA libraries were prepared following the manufacturer’s protocol and sequenced with a NovaSeq 6000 S1 Reagent kit v1.5.

### WES simple western blot

Following treatment, cells were lysed in RIPA buffer (Cell Signaling Technologies, USA, Cat. #9806) containing Halt protease and phosphatase inhibitor cocktail (Thermo Fisher, USA, Cat. #78430). Protein expression was analyzed using the Simple Western system (ProteinSimple, USA, Cat. #004-600) with a 12–230 kDa Separation Module (ProteinSimple, USA, Cat. #SM-W004). The following primary antibodies were used: Stat1 (Cell Signaling, Cat. #9172), phospho-Stat1 (Cell Signaling, Cat. #9167), β-actin (Cell Signaling, Cat. #5125), Stat2 (Cell Signaling, Cat. #72604), E-cadherin (Cell Signaling, Cat. #3195), RPE65 (Abcam, Cat. #ab231782), CRALBP (Abcam, Cat. #ab243664), and p16^INK4a^ (Abcam, Cat. #ab189034). Detection was performed using either the Anti-Rabbit Detection Kit (ProteinSimple, Cat. #DM-001) or the Anti-Mouse Detection Kit (ProteinSimple, Cat. #DM-002), depending on the primary antibody.

For sample preparation, RIPA-lysed cell lysates were quantified and diluted to 1 μg/μL in 5x Fluorescent Master Mix (ProteinSimple, USA, Cat. #PS-FL01-8), then heated at 95 °C for 5 min. Samples, along with Antibody Diluent (ProteinSimple, USA, Cat. #042-203), diluted primary and secondary antibodies, and chemiluminescent substrate, were loaded onto the WES capillary plate. The WES system was run with the following settings: stacking and separation at 375 V for 25 min, blocking for 5 min, primary and secondary antibody incubation for 30 min each, and chemiluminescence detection for 4 s.

### Histological analysis and immunohistochemistry

Paraffin sections (4 μm) of mouse eyes and human eyes (San Diego Eye Bank, San Diego, CA, USA) used for immunofluorescent detection of RPE65 and p16^INK4A^ underwent antigen retrieval with citrate buffer pH 6.0 (Invitrogen, Cat. #005000) at 98 °C for 10 min. After washing with PBS, the slides were incubated with primary antibodies for RPE65 (rabbit monoclonal, Abcam, Cat. #ab231782) (mouse monoclonal, Invitrogen, Cat. #MA1-16578), CRALBP (rabbit monoclonal, Abcam, Cat. #ab243664), p16^INK4a^ (rabbit polyclonal, Abcam, Cat. #ab189034), all diluted (1:100) in PBS with 0.2% Triton X-100 plus 10% donkey serum, and then incubated over night at 4 °C. Primary stained slides were visualized using an Alex Fluor-conjugated secondary antibody (1/200, Invitrogen, Cat. #A31572, A21422). All stained slides included negative controls for validation. Representative high-resolution images were generated by Zeiss LSM710 confocal microscope with Zen 3.3 black edition software (Zeiss, Germany).

Immunohistochemical detection of STING and IFN-β in human eyes employed a Ventana Discovery automated staining system using our previously reported techniques [101]. The control (*n* = 11) eyes and those with AMD (*n* = 11 for geographic atrophy; and *n* = 8 for disciform scars) were from autopsies performed at Duke University Hospital between 1991 and 2017, and the AMD stage was assigned using the criteria of Sarks [39] (Duke Health IRB protocol Pro00071222). Staining intensity was graded on a scale from 0 to 4 for the various cell types within each ocular tissue layer. For each cell type, the mean staining intensity along with the standard deviation (mean ± SD) was calculated. All grading of the staining intensity was performed using the same Olympus BX41 microscope (Evident Scientific, Inc., Waltham, MA, USA) to ensure consistency. In the case of IFN-β expression in macular choroidal melanocytes, a composite staining score was determined by multiplying the staining intensity by the percentage of cells expressing IFN-β expression. Photomicroscopy of human eye sections was conducted using an Olympus AH-3 microscope equipped with an AmScope MU1000 digital camera (AmScope, Irvine, CA, USA). All digital photomicrographs were processed uniformly using CyberLink PhotoDirector 365 (CyberLink.com Corporation, Los Angeles, CA, USA) with the auto color enhancer, denoise, deblur, and “as shot” white balance features to optimize image clarity and consistency.

H&E of *Il17a KI* mice: Mouse eyes were enucleated post-euthanasia, rinsed in 1X PBS, and trimmed of extraocular tissues. Samples were fixed in commercial Excalibur Pathology’s alcoholic fixative (Excalibur’s alcoholic Z-fix, Excalibur Pathology, Norman, OK, USA) at room temperature (RT) overnight. Following fixation, tissues were dehydrated through a graded ethanol series (70–100%), cleared in xylene (three changes, 30 min each), and infiltrated with paraffin at 60 °C (three changes, 60 min each). Tissues were embedded in paraffin blocks, and 10 μm sections were cut using a rotary microtome and mounted onto glass slides.

For H&E staining, sections were deparaffinized in xylene (three changes, 5 min each), rehydrated through a descending ethanol gradient (100%, 95%, 80%, 70%, 30%, water), and stained with Harris’ hematoxylin for 3 min. Slides were differentiated in 1% acid alcohol, blued in Scott’s tap water substitute, counterstained with Eosin Y for 1 min, dehydrated again in ascending series of ethanol gradient, cleared with xylene, and mounted with DPX mounting medium. All stained sections were imaged using an Olympus brightfield microscope at 20x and 60x (Oil), objective magnification.

### Cell senescence assay

Paraformaldehyde fixed (4%) eyes were embedded with Tissue-Tek® O.C.T. compound after a sucrose (30%) dehydration process. Fixed eyes were sectioned at 12 μm with a Leica Cryo-station. Senescent retinal cells were stained with a senescence detection kit (Abcam, Cat. #ab65351) following the manufacturer’s protocol. In short, the slides were brought to RT, then a hydrophobic pen was used to circle the tissue. After washing the tissue three times with PBS, the staining solution mix (Staining Solution, staining supplement, and 20 mg/mL X-Gal in DMSO) was added to each slide and then incubated at 37 °C for 12 h. After incubation, the slides were washed with PBS before mounting. Stained slides were imaged within 6 h using an Olympus VS120 scanner with OlyVia software (Olympus, Japan). Slide scans of all stains can be made available upon request. SA-β-Galactosidase positive cells of scanned retinas were annotated. The number of SA-β-Galactosidase positive cells and the tissue area were automatically calculated by the HALO^®^ AI^TM^ (Indica Labs v3.0.311.261). The number of SA-β-Galactosidase positive cells was normalized to the tissue area, and then the fold change between Null and IFN-β was calculated based on six individual eyes with two images per eye.

### RNAscope in-situ hybridization

To perform RNAscope in situ hybridization, mouse eyes were fixed in Modified Davidson’s Fixative (Electron Microscopy Sciences, Hartford, PA, Cat. #6413350) for 15 min at RT before an overnight fix with 4% (w/v) formalin (Avantor, Cat. #3933.9020) at 4 °C. After embedding, all samples were stored at 4 °C before staining. Formalin-fixed paraffin-embedded (FFPE) blocks were sectioned at 4 µm, with one section used for a positive probe, one for a negative probe, and the other sections used for the target probe (RNAscope®Probe-Mm-ifnb1-Mus mRNA, ACD, Cat. #406531). RNAscope in situ hybridization was performed according to the protocol of the RNAscope Multiplex Fluorescent Reagent Kit V2 (ACD, Cat. #323136). Briefly, FFPE sections were de-paraffinized and dried in a drying oven. RNAscope ® Hydrogen Peroxide was added to the slide for 10 min at RT. For the antigen retrieval process, slides were incubated at 100 °C for 15 min in a HistosPRO Rapid microwave histoprocessor (Milestone, Italy) with RNAscope® 1x Target Retrieval Reagent, then washed in water twice and dehydrated in 100% ethanol, and finally treated with RNAscope^®^ Protease Plus for 30 min at 40 °C. The C1 probe was detected in Opal^TM^ 570 (Akoya Biosciences, USA). Before mounting the slides, DAPI (Thermo Scientific, Cat. #62248) was added to label the nuclei.

### RPE cell culture

All cell culture experiments were performed using human retinal pigment epithelial (hRPE) cells. Cells used for western blot and WES analysis were obtained from ScienCell (Cat. #6540, Lot #12637) and cultured in RtEGM™ BulletKit medium (LONZA, Cat. #00195409), supplemented with RtEGM™ SingleQuots (Cat. #00195407), L-glutamine (Cat. #00557283), basic fibroblast growth factor (bFGF; Cat. #00557284), fetal bovine serum (FBS; Cat. #00557285), and GA1000 (Cat. #00557286). Unless otherwise specified, cells were maintained at 37 °C in a humidified incubator with 5% CO₂ and 90% relative humidity. T175 cell culture flasks were pre-coated with 5 μg/mL laminin-521 diluted in phosphate-buffered saline (PBS) containing Mg²⁺/Ca²⁺ for 60 min. The coating solution was aspirated prior to cell seeding. Frozen hRPE cells were thawed in a 37 °C water bath for 1–2 min and transferred into 35 mL of pre-warmed Growth Medium 1 (see Table 1), followed by plating into the coated T175 flask. Cells were expanded for 3 additional days until they reached at least 80% confluency, then passaged into appropriate culture vessels. Before passaging, all vessels were coated with 5 μg/mL laminin-521 in PBS (Mg²⁺/Ca²⁺), and the coating solution was aspirated before adding cells. Growth Medium 1 was aspirated, and cells were washed with 20 mL PBS, followed by dissociation using 10 mL of 0.05% trypsin-EDTA for 3–5 min at 37 °C. Enzymatic activity was quenched by adding 10 mL of Growth Medium 2 (Table 1), and cell concentration was measured using an automated TC20™ cell counter (Bio-Rad, USA). Cells were cultured in Growth Medium 2 for 4 days with a medium change on day 2. On day 4, once the culture reached full confluency (100%), the medium was replaced with serum-deprived maturation medium (Table 1). Cells were maintained in maturation medium for 17 days, with medium changes every 3–4 days. By day 21 post-passaging, hRPE cells exhibited a cobblestone-like morphology with strong pigmentation and were considered mature for downstream applications.

**Table 1:** Composition of cell culture media.

Item	Volume (mL)
Growth media 1	
Epithelial cell medium (ScienCell)	500
FBS	10%
Epithelial cell growth supplement	5
Antibiotic solution	5
Growth media 2	
RPE basal media (Lonza)	200
L-glutamine	4
h-FGF-B	1
FBS	4%
GA-1000	0.2
Maturation medium	
RPE basal medium (Lonza)	200
L-glutamine	4
hFGF-B	1
GA-1000	0.2

Study cohort and clinical assessments: To examine AMD-associated morphological and pathological changes-including pigmentary alterations, drusen deposition, and GA, we performed comprehensive clinical evaluations of sibling pairs (with and without AMD). These assessments were designed to correlate drusen accumulation and RPE changes in each patient with atrophic AMD and their unaffected siblings (Retina Foundation of the Southwest, Dallas, Texas; Protocol number: ARMD-SS-2019; IRB tracking number: 520190103). Phenotypic characterization of AMD features, such as subretinal drusenoid deposits (SDD) and complete RPE and outer retinal atrophy (cRORA), was evaluated using spectral-domain optical coherence tomography (SD-OCT) and fundus autofluorescence (FAF) imaging with the Heidelberg Spectralis HRA + OCT (Heidelberg Engineering, Germany). Swept-source OCT angiography (OCT-A; Elite 9000, Carl Zeiss AG, Germany) was additionally employed to exclude vascular abnormalities. All imaging modalities complemented standard fundus examinations. Patients who met predefined inclusion and exclusion criteria were enrolled in the study. Specifically, we included individuals with intermediate to advanced stages of dry AMD, characterized by the presence of hyperreflective foci and drusen. The inclusion criteria were based on a confirmed diagnosis of atrophic AMD, as determined by OCT and fundus imaging. After through clinical examination, 20–30 mL of peripheral whole blood was collected from each participant for peripheral blood mononuclear cell (PBMC) isolation and subsequent induced pluripotent stem cell (iPSC) reprogramming.

### Peripheral blood mononuclear cell (PBMC) isolation

PBMCs were obtained from whole blood samples collected in Vacutainer^®^ CPT™ tubes (BD Biosciences, Cat. #14-959-51D) from intermediate to late AMD patients (*n* = 3) and their unaffected siblings (*n* = 3). To ensure proper mixing of the blood with the anti-coagulant and density gradient medium, the tubes were gently inverted 8–10 times. Subsequently, samples were centrifuged at room temperature (RT) for 20 min at 1600 × *g* using a swing-bucket rotor. Following centrifugation, the upper plasma layer was aspirated, and the PBMC interphase was carefully collected and transferred to a 15 mL conical tube. The cells were then diluted with chilled Iscove’s Modified Dulbecco’s Medium (IMDM; Cytiva, Cat. #SH30259.01) to a final volume of 15 mL and centrifuged at RT for 5 min at 300 × *g*. After discarding the supernatant, the cell pellet was resuspended in IMDM for cell counting using a hemocytometer or automated cell counter. Finally, PBMCs were cryopreserved at a density of 4–5 × 10⁶ cells/mL in CryoStor CS10 (StemCell Technologies, Cat. #07930) using a controlled rate freezing container before long-term storage in liquid nitrogen and iPSC reprogramming. The cells were genotyped for *CFH* Y402H or Y402 polymorphism by Taqman probe *CFH* Y402H polymorphism (SNP ID: rs1061170) assay (Invitrogen, USA).

### Induced pluripotent stem cell (iPSCs) reprogramming

The iPSC generation protocol was modified from an established method [102]. Briefly, cryopreserved PBMCs (5 × 10^6^ cells) were thawed and cultured for six days in StemSpan™ using the Human CD34^+^ Cell Nucleofector™ Kit (Lonza, Cat. #VPA-1003) with the following episomal plasmids (2 μg each): pCXLE-hOCT3/4-shp53-F (Addgene, Cat. #27077; encoding OCT3/4 and TP53 shRNA), pCXLE-hUL (Addgene, Cat. #27078; L-MYC and LIN28), pCXLE-hSK (Addgene, Cat. #27080; SOX2 and KLF4) and pCXWB-EBNA1 (Addgene, Cat. #37624; EBNA1). Nucleofection was performed using the Lonza 2b Nucleofector system (program U-008). Post-nucleofection, cells were plated on iMatrix-511 in mTeSR Plus medium (StemCell Technologies, Cat. #100-0276) with the same cytokine cocktail. Fresh mTeSR Plus (without cytokines) was supplemented every 48 h. On day 8, the medium was fully replaced with mTeSR™ Plus and refreshed every other day thereafter. By day 14, iPSC colonies were manually isolated and expanded on Matrigel-coated plates in mTeSR Plus. For passaging, cells were dissociated using ReLeSR (StemCell Technologies, Cat. #100-0483) and cryopreserved at passage 5 in CryoStor CS10. For routine expansion, iPSCs were enzymatically dissociated into single cells (Accutase; Sigma, Cat. #A6964) and maintained at 37 °C, 5% CO_2_, and 21% O_2_.

### hiPS-Retinal pigment epithelium (RPE) differentiation

hiPSCs derived from an AMD patient and a healthy sibling were differentiated into RPE cells using an established method [45]. Prior to differentiation, hiPSCs were cultured on growth factor-reduced Matrigel (Corning, Cat. #354230) in mTeSR1 plus medium under 5% CO₂ and passaged as clonal colonies using 5 µM blebbistatin (Sigma, Cat. #B0560). To initiate differentiation, hiPSCs were seeded at 30,000 cells/cm² and grown to confluence in mTeSR Plus medium. The medium was then switched to RPE differentiation medium, consisting of DMEM/F12 (Gibco, Cat. #11320033) supplemented with 15% knockout serum replacement (Gibco, Cat. #10828028), 2 mM L-glutamine (Gibco, Cat. #25030081), 1% non-essential amino acids (Gibco, Cat. #11140050), 0.1 mM β-Mercaptoethanol (Sigma, Cat. #444203), 1% antibiotic-antimycotic (Gibco, Cat. #15240) and 10 mM nicotinamide (Sigma, Cat. #72340) and maintained in this medium for approximately 50 days, with regular medium changes. Emerging RPE cells were then enzymatically dissociated using 0.25% collagenase IV (Gibco, Cat. #17104019) and further dissociated into single-cell suspensions using AccuMAX (Sigma, Cat. #A7089). The dissociated cells were replated onto fresh matrigel-coated plates and cultured in RPE maintenance medium (70% DMEM (Gibco, Cat. #11965092), 30% Ham’s F12 (Gibco, Cat. #11765054, 2% B-27 serum free supplement (Gibco, Cat. #17504044) and 1% antibiotic-antimycotic (Gibco, Cat. #15240062) for an additional 2–3 months to allow maturation into functional RPE monolayers. For IFNAR1 blockade experiments, 100 nM of anifrolumab (Medchem Express; Cat. No.: HY-P99168) was used to treat polarized RPE cells in transwell insert for 24 h.

### Collection of conditioned media from polarized iPSC-RPE cultures

Conditioned media were collected from polarized RPE monolayers derived from an AMD patient and an unaffected sibling and cultured in a 12-well transwell system. Media from apical (0.5 mL/well) and basal (1.5 mL/well) compartments were pooled separately for each condition. To remove cellular debris, the media were centrifuged at 300 × *g* for 10 min at 4 °C. The supernatants were carefully collected, leaving 200–300 μL to avoid disturbing the pellet. Cleared media was stored at −80 °C.

### ELISA for IFN-β and IL-17A quantification

Secreted levels of human interferon-beta (IFN-β) and interleukin-17 (IL-17) in the iPSC-RPE conditioned media were measured using Quantikine ELISA kits (R&D Systems, Cat. #DIFNB0 for IFN-β; Cat. #D1700 for IL-17A) according to the manufacturer’s instructions. Briefly, 96-well plates pre-coated with capture antibodies were washed and blocked as per kit protocols. Standards and samples were added in triplicate (50 μL per well) and incubated for 2 h at RT. After extensive washing, 200 μL of detection antibody conjugation was added and incubated for an additional 2 h at RT on the shaker. Wells were washed, and 200 μL of substrate solution was added, followed by incubation in dark for 30 min. The reaction was stopped by adding 50 μL of stop solution. Absorbance was measured at 450 nm with wavelength correction at 570 nm using a microplate reader (BioTek Synergy). Cytokine concentrations were determined by plotting sample absorbance values against a standard curve generated from known concentrations of recombinant human IFN-β and IL-17A proteins.

### Generation of human retinal organoids

An hiPSC line derived from CD34+ cord blood was used in this study (Thermo Fisher Scientific, Cat. #A1894546) [102]. This cell line was verified to have a normal karyotype and was free of contamination. Undifferentiated hiPSCs and derived retinal organoids were routinely tested for Mycoplasma contamination by PCR. Briefly the hiPSCs [102] were enzymatically dissociated into small clumps and cultured in mTeSR1 medium and 10 mM Blebbistatin (Sigma, Cat# 1177356-70-5) to induce aggregate formation. Aggregates were gradually transitioned into neural-induction medium (NIM) on day 3. On day 7, aggregates were seeded onto Matrigel-coated dishes containing NIM at an approximate density of 20 aggregates per cm^2^. On day 16, NIM was replaced with DMEM/F12 (3:1) supplemented with 2% B27 (without vitamin A, Invitrogen), 1x NEAA, and 1% antibiotic–antimycotic (Gibco). During the fourth week of differentiation, the horseshoe-shaped neural retina was manually detached with a sharpened tungsten needle, collected, and cultured in suspension at 37 °C in a humidified 5% CO_2_ incubator, where they gradually formed 3D retinal organoids. To promote photoreceptor maturation, suspension cultures of retinal organoids were supplemented daily with 1 mM all-trans retinoic acid (Sigma, Cat# 302-79-4) during weeks 7-17. Retinal organoids at D180 were used for the experiments. Human retinal organoids were exposed to different conditioned hiPSC-RPE cell culture media for 72 h: i. apical media from hiPSC-RPE derived from an AMD patient; ii. basal media from hiPSC-RPE derived from an AMD patient; iii. apical media from hiPSC-RPE derived from a healthy control; iv. basal media from hiPSC-RPE derived from a healthy control; v. apical media from hiPSC-RPE derived from an AMD patient pre-treated with antibodies to neutralize IFN-β; and regular RPE cell culture media supplemented with recombinant IFN-β.

### Immunofluorescence

For immunofluorescence performed on human retinal organoids, samples were fixed in 4% paraformaldehyde for 1 h, washed twice in PBS, and cryoprotected with a sucrose gradient (6.75, 12.5, and 25%, overnight at 4 °C) with a final incubation in 25% sucrose/OCT (2:1 ratio respectively) for 1 h at RT. Samples were embedded in 25% sucrose/OCT Tissue-Tek (Sakura), frozen, and stored at −80 °C until used. Cryosections of 12- to 16-μm thickness were obtained and collected on Superfrost Plus slides. Sections were air-dried for 1 h, washed three times in PBS, blocked in 10% goat serum in PBS with 0.25% Triton X-100 for 1 h at RT, and incubated overnight with primary antibodies for CRALBP, recoverin, cone arrestin, and rod opsin diluted (1:100) in 2% goat serum in PBS with 0.05% Triton X-100 at 4 °C. The next day, the slides were washed three times in PBS and incubated with an Alexa Fluor–conjugated secondary antibody (1:500; Molecular Probes) in PBS for 1 h in the dark at RT. The slides were then washed three times in PBS, incubated in DAPI (1:1000 in PBS) for 10 min, and cover-slipped using DAKO fluorescent mounting medium. Fluorescence images were acquired with a Nikon C2 laser scanning confocal microscope (Melville, NY, USA). The images were minimally processed using Adobe Photoshop CS5 (San Diego, CA, USA).

### TUNEL assay

Fixed human retinal organoids were stained using an in situ cell death detection kit conjugated with fluorescein isothiocyanate (Roche) according to the manufacturer’s instructions. For controls, the terminal deoxynucleotidyl transferase enzyme was either omitted from the labeling solution (negative control) or a human retinal organoid was incubated with 1000 U/ml DNase I recombinant for 10 min at RT to induce DNA strand breaks prior to labeling procedures (positive control). Fluorescence images were acquired with a Nikon C2 laser scanning confocal microscope (Melville, NY, USA).

### Lactate dehydrogenase cytotoxicity assay (LDH)

Retinal organoids were exposed to conditioned media collected from iPSC-derived RPE cells from an AMD patient, sibling control RPE cells, recombinant IFN-β, or media supplemented with neutralizing IFN-β antibodies. Treatments were applied for 0, 24, 48, or 72 h. Following treatment, 50 μL of culture supernatant was collected from each well and transferred to a fresh 96-well plate for lactate dehydrogenase (LDH) quantification. The assay was performed using the CytoTox 96^®^ Non-Radioactive Cytotoxicity Assay Kit (Promega, Cat. #G1780), according to the manufacturer’s instructions. Briefly, 50 μL of supernatant was mixed with 50 μL of CytoTox 96^®^ Reagent in each well, and the plate was incubated for 30 min at room temperature (RT) in the dark. The reaction was terminated by adding 50 μL of Stop Solution, and absorbance was measured at 490 nm using a microplate reader. A positive control for maximum LDH release was included as provided in the kit. All experiments were conducted in biological triplicate, each with technical triplicates. Cytotoxicity was calculated using the following formula: Percent cytotoxicity = 100 × [Experimental LDH release (OD₄₉₀)/Maximum LDH release (OD₄₉₀)].

### RNA isolation and purification

Following treatment, medium was aspirated from human RPE (hRPE) cultures (media was retained separately for Luminex assays), and cells were gently washed once with PBS. To each well of a 6-well plate, 200 μL of cold RNA isolation tissue lysis buffer was added. Plates were placed on an orbital shaker at 1200 rpm for 5 min to facilitate cell lysis. Lysates were collected using a cell scraper and immediately transferred to a 96-well processing cartridge kept on ice. RNA was isolated and purified using the MagNA Pure 96 Cellular RNA Large Volume Kit (Roche, Cat. #05467535001) on the MagNA Pure 96 System (Roche), according to the manufacturer’s protocol. RNA was eluted in 50 μL of nuclease-free H₂O. Concentrations were determined using a NanoDrop Eight UV-Vis spectrophotometer (Thermo Fisher Scientific), and samples were adjusted to equimolar concentrations and stored at –80 °C until further use.

### cDNA synthesis

RNA samples were thawed on ice and reverse-transcribed into cDNA using the iScript cDNA Synthesis Kit (Bio-Rad) according to the manufacturer’s instructions, in a final reaction volume of 20 μL per sample. Reverse transcription was performed in a thermal cycler with the following cycling conditions: priming at 25 °C for 5 min, reverse transcription at 46 °C for 20 min, and enzyme inactivation at 95 °C for 1 min. Synthesized cDNA was stored at 4 °C.

### qRT-PCR

Gene expression analysis was performed using quantitative real-time PCR (qRT-PCR). A master mix was prepared as described in Table 2. For each reaction, 8 μL of master mix was combined with 2 μL of cDNA and loaded into a 384-well PCR plate kept on ice. Plates were briefly centrifuged to eliminate air bubbles before being placed into the QuantStudio 12 K Flex Real-Time PCR System (Thermo Fisher Scientific, USA). Reactions were run using standard TaqMan comparative Ct settings.

**Table 2:** qPCR master mix.

Component	Volume/reaction (µL)
2X TaqMan Master mix	5
H_2_O	2.33
60x TaqMan probe VIC (GUSB gene)	0.17
20x TaqMan probe FAM (gene of interest)	0.50

Relative gene expression was quantified using the ∆∆Ct method. The housekeeping gene GUSB was included in each reaction as an internal reference. ∆CT values were calculated within each well to account for technical variability, such as pipetting inconsistencies.

### Luminex assay

To determine cytokine concentrations of cell conditioned media, a Luminex assay with cell supernatants was performed. 1 mL of cell media of treated mature hRPE cells was collected and 41.7 μL of 25x protease inhibitor cocktail (1 tablet + 2 mL of PBS) was added to prevent cytokine degradation. Supernatants were stored at −80 °C. Cell supernatants were slowly thawed on ice or at 4 °C. Standards were prepared in calibrator diluent RD6-52 according to the respective certificate of analysis provided with each assay kit. Standard cocktail solution was used to produce a 4-fold dilution series (7 dilutions). 50 μL of standards and sample media were added in duplicates to each well of the 96-well assay plate. 50 μL of diluted microparticle cocktail (500 μL + 5 mL diluent RD2-1) was added and incubated for 2 h at RT and 1200 rpm on an orbital shaker. The plate was washed 3 times with was buffer (900 mL water + 100 mL 10x PBS + 250 mg Tween 20) using a magnetic microplate washing system. Subsequently, 50 μL of diluted biotin-antibody cocktail (500 μL + 5 mL diluent RD2-1) were added and incubated for 1 h at RT and 1200 rpm. The plate was washed 3 times as before, 50 μL of diluted streptavidin-PE (220 μL + 5.35 mL wash buffer) was added to each well, and incubated for 30 min at RT and 1200 rpm. After 3 washing steps, 100 μL of was buffer wear added to each, incubated for 2 min at RT and 120 rpm and then measured using the Luminex FlexMAP 3D system (Luminex Corporation, Austin, USA). Initial analysis was performed with xPONENT software (version 4.3). Standard curves were calculated using the 5-parameter logistic regression weighed by 1/Y2.

### Western blot

Protein lysates were extracted from mouse RPE tissues and photoreceptor organoids using 1X RIPA lysis buffer supplemented with phosphatase and protease inhibitors. A total of 15 μg of protein per sample was loaded onto a 4–12% Bis-Tris SDS-PAGE gel and resolved by electrophoresis. Proteins were then transferred to PVDF membranes using the iBlot™ dry transfer system (Thermo Fisher Scientific). Membranes were blocked with 3% BSA in 1X TBST (Tris-buffered saline with 0.1% Tween-20) for 1 h at RT, followed by overnight incubation with primary antibodies at 4 °C on a shaker. The following primary antibodies were used to probe the membrane; anti-CRYBA1 (1:1000, Invitrogen, Cat. #PA5-28954), anti-STING (1:1000, Novus Biologicals, Cat. #NBP2-24683), anti-IL17A (1:1000, Abcam, Cat. #ab79056), anti-IFNAR1 (1:1000, Proteintech, Cat. #83002-4-RR), anti-phospho STAT1 (1:1000, Abcam, Cat. #109461), STAT1 (1:1000, Cell Signaling Technology, Cat. #14994), anti-phospho STAT3 (Tyr705) (1:1000, Cell Signaling Technology, Cat. #9131), anti-STAT3 (1:1000, Cell Signaling Technology, Cat. #4904), anti-cleaved caspase 1 (1:1000, Cell Signaling Technology, Cat. #4199), anti-H3 (1:2000, Cell Signaling Technology, Cat. #4499, Cell Signaling Technology), anti-β-Actin (1:2000, Cat. #4970, Cell Signaling Technology) and anti-Vinculin (1:2000, Cell Signaling Technology, Cat. #13901). After primary incubation, membranes were washed three times in TBST for 10 min each and incubated with HRP-conjugated anti-rabbit IgG secondary antibody (1:3000, Cell Signaling Technology, Cat. #7074) for 1 h at RT with gentle shaking. Membranes were washed again three times with TBST and developed using enhanced chemiluminescence (ECL; Azure Biosystems). Images were captured using the Azure gel documentation system.

### Seahorse XF MitoStress test

For the Seahorse assay, 2500 cells/well of hRPE cells were plated in 0.15 mL/well in a Seahorse 96-well plate. Mature hRPE cells that had been treated for 96 h were washed once with warm assay medium (47.4 mL DMEM, 0.6 mL glucose 1.0 M, 0.5 mL glutamine 200 mM, 1.0 mL pyruvate 100 mM) and then incubated at 37 °C and 0% CO_2_ for 60 min. Stock solutions of oligomycin, FCCP, and antimycinA/rotenone were prepared with assay medium according to the manufacturer’s instructions. Oligomcyin was loaded in port A, FCCP in port B, and antimycinA/rotenone in port C. Measurements were performed with the Seahorse XFe96 analyzer (Agilent Technologies, Santa Clara, USA) and the default XF Cell Mito Stress Test assay template. A loaded sensor cartridge with the utility plate containing calibration solution was loaded for calibration. Assay medium of hRPE cells was replaced with 180 μL of fresh medium per well and then loaded in the Seahorse analyzer to run the assay. Final calculations of assay parameters were performed with Wave software (Agilent Technologies, Santa Clara, USA) and its integrated report generator.

### Bioinformatics analysis of scRNA-seq data from retinal tissue

Single-cell RNA sequencing (scRNA-seq) data were analyzed using the besca package [100], which is built upon the scanpy framework [101]. Cell clusters were annotated based on established retinal cell type markers (Fig. 3b, c). Notably, a distinct subset of cells adjacent to the Müller glia cluster was observed exclusively in IFN-β-treated samples. This population was labeled as “reactive Müller cells” based on elevated expression of GFAP and LCN2, indicating a reactive phenotype. For differential expression analysis, we compared cells of the same annotated type across treatment conditions using the Wilcoxon rank-sum test as implemented in scanpy’s rank_genes_groups function. (Reactive and non-reactive Müller cells were grouped together for this analysis, as they represent condition-dependent states of the same lineage.) The resulting test statistics—positive for upregulated genes and negative for downregulated genes—were then used as input for gene set enrichment analysis (GSEA) using the fgsea package (https://bioconductor.org/packages/fgsea).

### Bioinformatics analysis of bulk RNA-seq data

Raw sequence data quality was first assessed, followed by adapter and low-quality base trimming using Trimmomatic v0.36. Trimmed reads were aligned to the Mus musculus reference genome (ENSEMBL) using the STAR aligner v2.5.2b, a splice-aware aligner capable of identifying and incorporating splice junctions to improve alignment accuracy. The alignment process generated BAM files. Gene-level quantification was performed using feature Counts from the Subread package v1.5.2, counting only uniquely mapped reads located within annotated exons. The resulting gene count matrix was used for downstream differential gene expression analysis with DESeq2. Comparisons between experimental groups were performed using the Wald test to compute p-values and log₂ fold changes. Genes with an adjusted *p*-value < 0.05 and absolute log2 fold change > 1 were considered differentially expressed.

### Statistical analyses

All statistical analyses were conducted using GraphPad Prism v8.2.0 (GraphPad Software, USA). Data are reported as mean ± standard deviation (SD). Normality of each dataset was assessed prior to hypothesis testing. For comparisons between two groups (e.g., cGAMP or IFN-β-treated vs. control), two-tailed unpaired Student’s *t*-tests were applied to datasets that passed the normality test. Statistical significance was defined as *p* < 0.05. For comparisons involving more than two groups, one-way or two-way ANOVA was used, followed by appropriate post hoc multiple comparison corrections.

## Supplementary information

Supplementary Figures and Legends

Raw data

## Data Availability

Sequencing data have been deposited in the NCBI GEO database under the following accession numbers: GSE313587 (bulk RNA sequencing) and GSE314326 (single-cell RNA sequencing). Raw data for the manuscript have been provided as Supplementary Material.
