# Supplementary material for: STING activation induces polarized cytokine secretion of IFN-β and IL-17A promoting photoreceptor death and choroidal disruption in age-related macular degeneration
Source: Cell Death Dis. 2026 Feb 27;17(1):283. doi: 10.1038/s41419-026-08491-w (PMC13031871; doi:10.1038/s41419-026-08491-w)
Supplement: Supplementary file 1 — Supplementary Figures and Legends [file 41419_2026_8491_MOESM1_ESM.pdf]

Supplementary Figure 1: **Prolonged IFN-β signaling causes retinal degeneration**

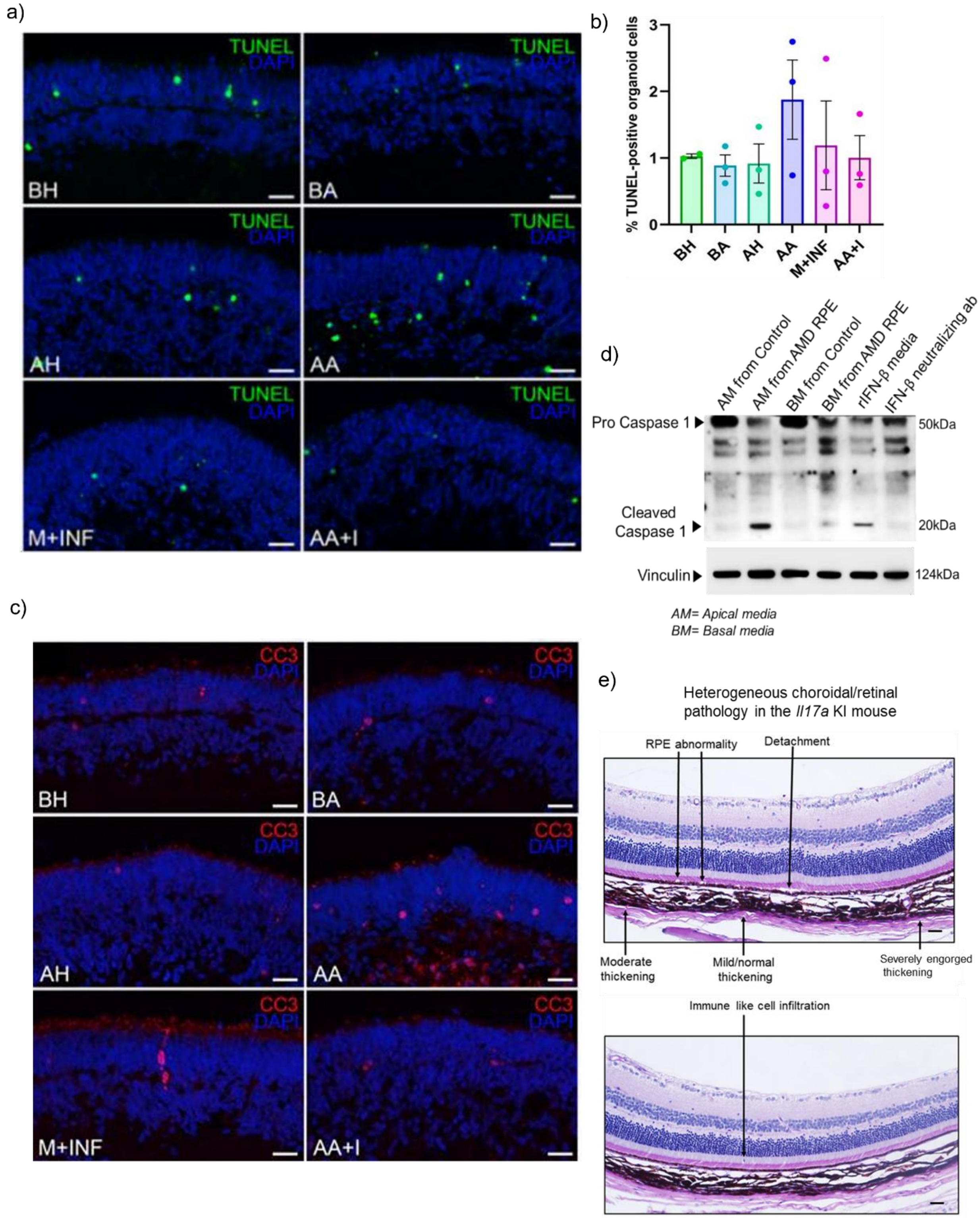

<u>Figure S1</u>: Prolonged IFN-β signaling causes photoreceptor death and retinal degeneration. a) TUNEL staining of retinal organoids after 72 h of treatment with conditioned media: BH (basal media from healthy individuals), BA (basal media from AMD patients), AH (apical media from healthy individuals), AA (apical media from AMD patients), M+INF (regular media with recombinant IFN-β), and AA+I (apical media from

AMD patients pretreated with IFN-β antibodies). b) Quantification of TUNEL-positive cells in retinal organoids after 72 h of treatment (n = 3). c) Immunofluorescence showing cleaved caspase-3 (CC3) expression in retinal organoids treated with conditioned media. Abbreviations: DAPI, 4′,6-diamidino-2-phenylindole; CC3, cleaved caspase-3; d) Western blot showing increased pyroptotic cleaved caspase-1 expression in D180 hiPSC-derived retinal organoids upon exposure to apical media from human AMD iPSC-RPE or media supplemented with recombinant IFN-β. Neutralization with anti-IFN-β antibody prevented the increased cleaved caspase-1 expression upon exposure to apical media from human AMD iPSC-RPE media. e) H&E staining of retinal tissue showing the heterogenous choroidal and retinal pathology observed in the *Il17a*-KI mice

Supplementary Figure 2: **Gene set enrichment analysis identified an upregulation of type I (GSEA) interferon in all retinal cells**

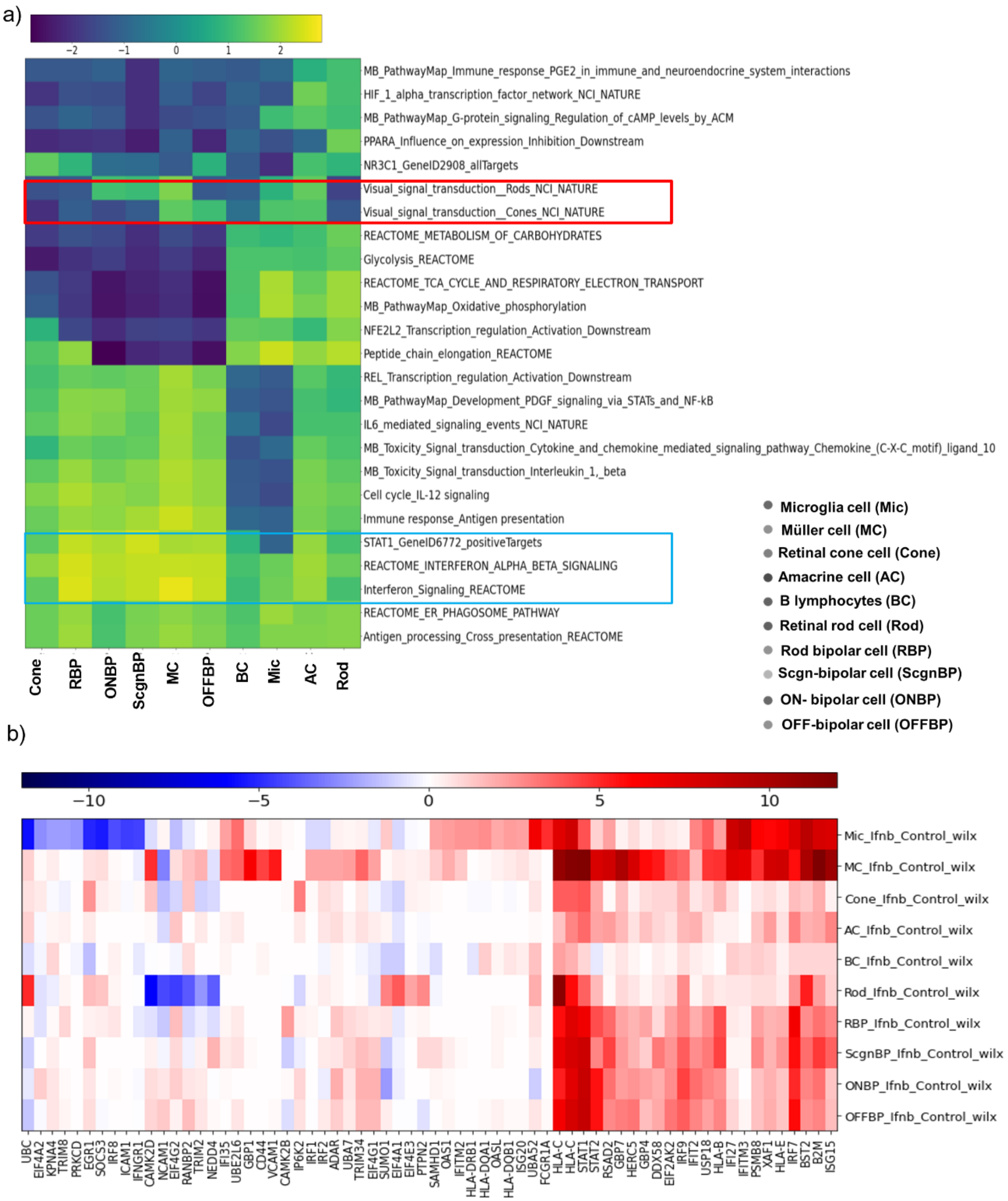

<u>Figure S2</u>: GSEA indicated a global upregulation of type I interferon responses in all retinal cells. a) Among REACTOM enriched pathways, interferon signaling was upregulated across annotated retinal cells (blue box). Down-regulated visual signaling (red box) was observed in photoreceptors, bipolar and B cells (n=4). b) The heat map of wilcoxon test statistical values genes in the interferon signaling network were upregulated across retinal cells, especially in Müller and bipolar cells (n=4).

Supplementary Figure 3. **scRNAseq analysis showed visual transduction genes reduced in rods and Müller cells of IFN-β -AAV2 induced (10 weeks) mouse eyes.**

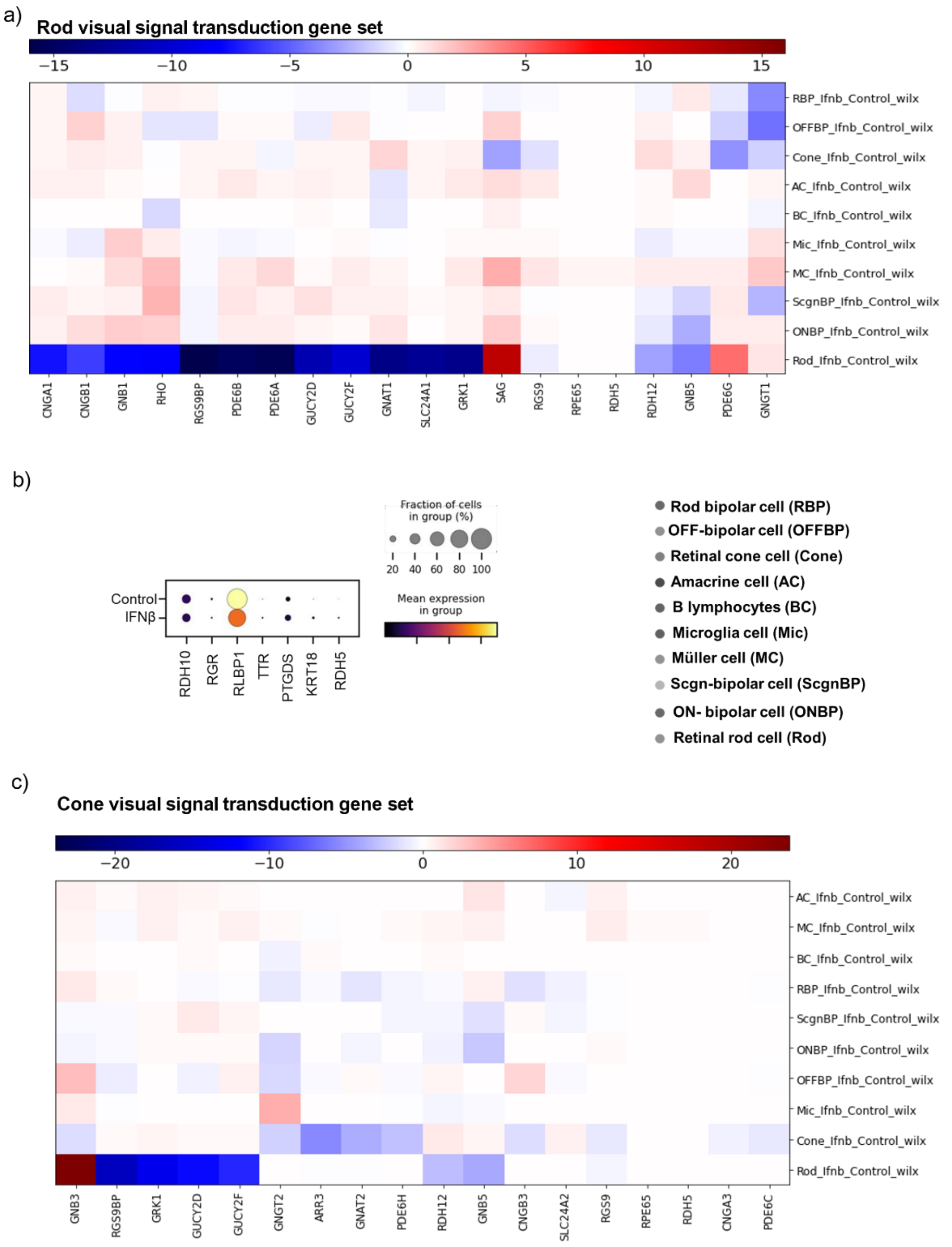

Figure S3: **scRNA-seq analysis revealed a reduction in visual transduction gene expression in rod photoreceptors and Müller glia of IFN-β-AAV2–treated mouse eyes at 10 weeks**. a) Rod visual signal transduction genes, including *RPE65* and *RDH5*, displayed in a heat map of Wilcoxon test statistical values, showed lower expression in rods of IFN-β-AAV2 induced eyes. b) Müller cell group from the scRNA analysis (Fig. 4b) shows a reduction of *CRALBP1/RLBP1* gene in IFN-β-AAV induced eyes compared with Null-AAV

eyes; however, no changes were observed in other retinal metabolism genes. c) Cone visual signal transduction gene set showed minimal changes with IFN-β stimulation, indicating a rod-specific response upon IFN-β induction in RPE (n=4).

Supplementary Figure 4. **Chronic IFN-β expression in mouse eyes leads to significant RPE degeneration by 20 weeks**

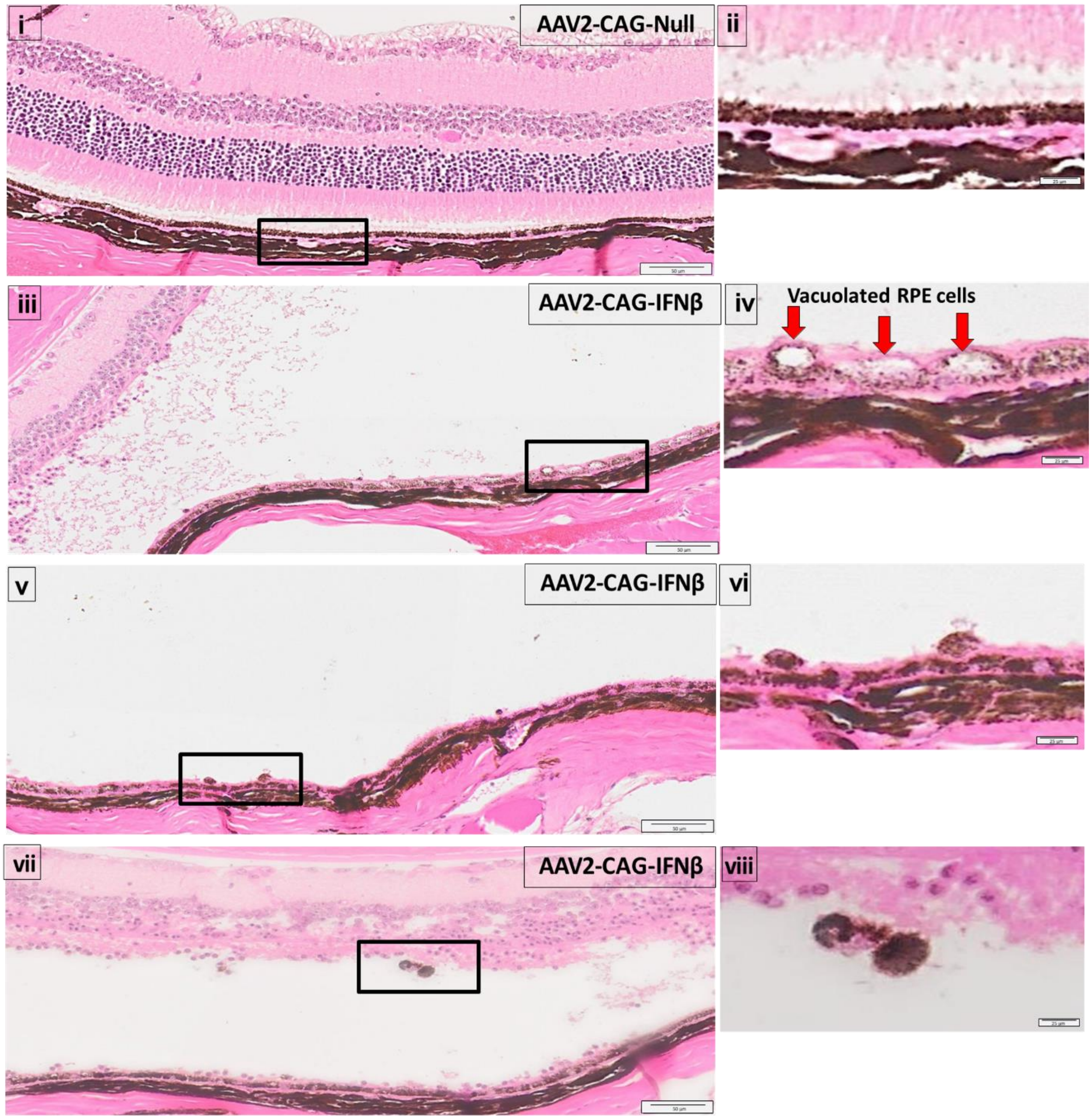

__Figure S4__: **H&E staining displayed profound retinal and RPE degeneration in IFN-β induced mouse eyes at 20 weeks post AAV injection**. a) Comparing with AAV2-CAG-Null mouse eyes, AAV2-CAG-IFN-induced eyes demonstrated degeneration of the photoreceptor layer (i, ii). AAV2-CAG-IFN induced eyes showed swollen RPE cells with vacuolated cytoplasm (iii, iv), pigment-laden cells attached to the surface of RPE cells (v, vi) and (vii, viii) and in the sub-retinal space (vii, viii) and intra-retinal microcysts (n=5 per group). Scale bar= 50 μm (Zoomed insets= 25 μm).

Supplementary Figure 5. **Chronic IFN-β stimulation of primary human RPE (7 days) reduces RPE cellularity, inflammation, increases senescence and its pharmacological inhibition lowers key NF-κB-driven proinflammatory cytokines.**

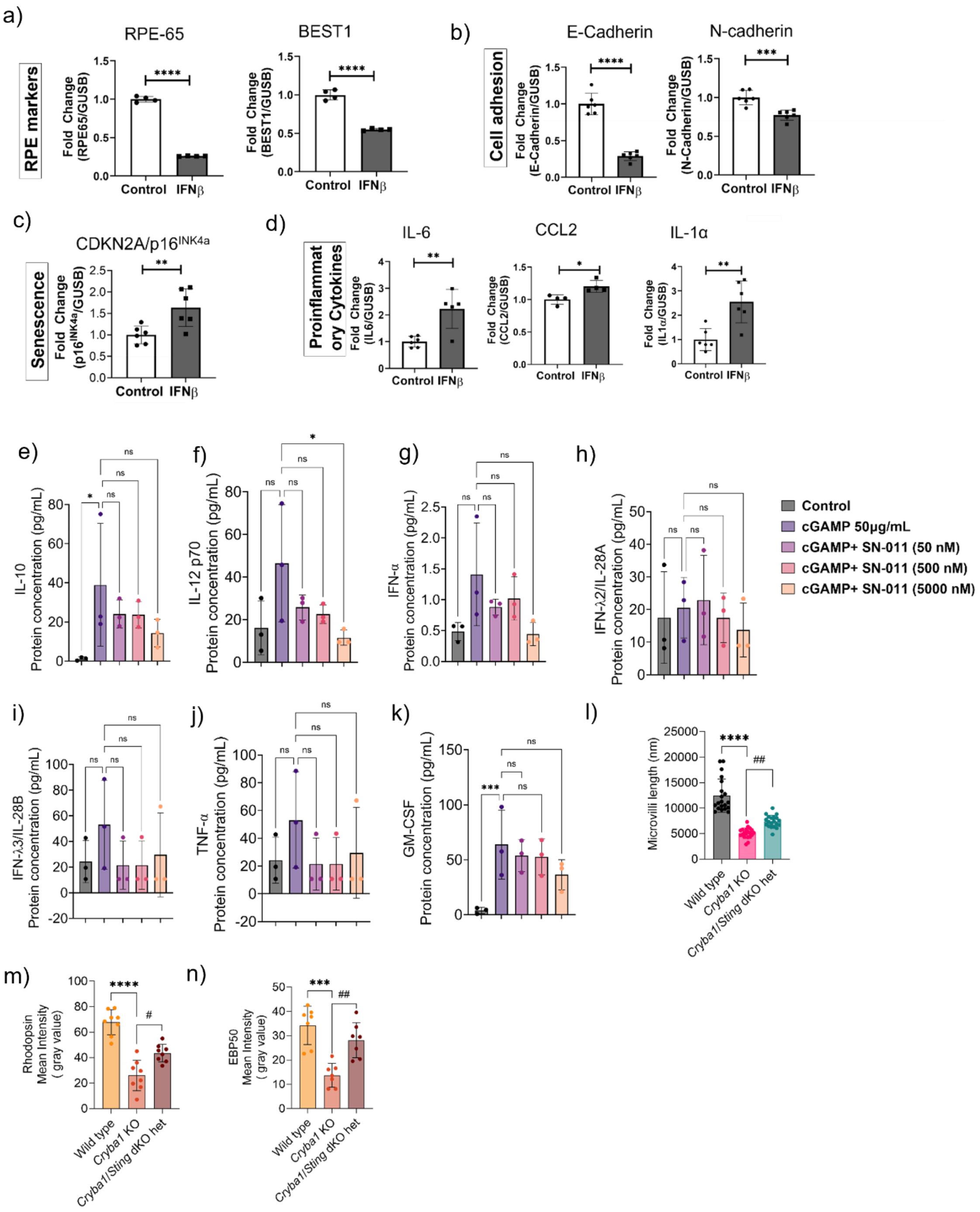

<u>Figure S5</u>: Chronic IFN-β stimulation of primary human RPE cells for 7 days leads to reduced RPE cellularity, increased senescence, and enhanced secretion of senescence-associated secretory phenotypes (SASP). a) Gene expression analysis of human RPE cells exposed to IFN-β (5000 IU/mL) for 7 days revealed a

reduction in RPE-specific markers *RPE65* and *BEST1* (n=4). b) Alterations in cell adhesion molecule expression were observed, with decreased E-cadherin and N-cadherin levels upon IFN-β induction (n=6). c) Expression of the senescence marker CDKN2A/p16^{INK4a} was elevated (n=6). d) Proinflammatory cytokines IL-6, CCL2, and IL-1α were upregulated. Mature human RPE (hRPE) cells were treated with 50 µg/mL cGAMP and varying concentrations of the STING inhibitor SN-011 for 24 h (n=6). Supernatants were collected and cytokine levels (pg/mL) were quantified using a Luminex assay: e) IL-10, f) IL-12p70, g) IFN-α, h) IFN-λ2/IL-28A, i) IFN-λ3/IL-28B, j) TNF-α and k) GM-CSF. cGAMP increased the levels of all cytokines, and the effect of cGAMP was blunted by the STING inhibitor SN-011.Values represent mean ± s.d from n=3 independent experiments. Statistical analyses were performed using an unpaired *t*-test (for comparisons between two groups) or two-way ANOVA with Dunnett's multiple comparisons test (for more than two groups). ns= not significant. *$p < 0.05$, **$p < 0.01$, ***$p < 0.001$, ****$p < 0.0001$. l) Bar graphs represent phalloidin microvilli length in Wild type, *Cryba1* KO and *Cryba1/Sting* dhet retinal sections, which was significantly lower in *Cryba1* KO mice, compared to wild type. But was rescued in age-matched dhet mice, relative to *Cryba1* KO, indicating rescue of structural changes in the dhet retina. n=3. mean microvilli length measured at six regions per section ± s.d. (n = 3). Statistical analysis was performed using the Kruskal–Wallis test. ****$p < 0.0001$, ## $p < 0.01$. Mean immunofluorescence intensity of m) rhodopsin n) EBP50 measured in Wild type, *Cryba1* KO and *Cryba1/Sting* dhet retinal sections shows significant rescue in the levels of both proteins in dhet retina compared to *Cryba1* KO, indicating towards rescue of both photoreceptor loss and RPE apical changes upon partial *Sting* deletion and *Cryba1* upregulation. Values represent the mean intensity ± s.d. (n = 3). Statistical analysis was performed using one-way ANOVA with Dunnett's multiple comparisons test. ****$p < 0.0001$, ***$p < 0.001$, # $p < 0.05$.
