# Supplementary material for: STING activation induces polarized cytokine secretion of IFN-β and IL-17A promoting photoreceptor death and choroidal disruption in age-related macular degeneration
Source: Cell Death Dis. 2026 Feb 27;17(1):283. doi: 10.1038/s41419-026-08491-w (PMC13031871; doi:10.1038/s41419-026-08491-w)

Raw immunoblots for Figure 6c

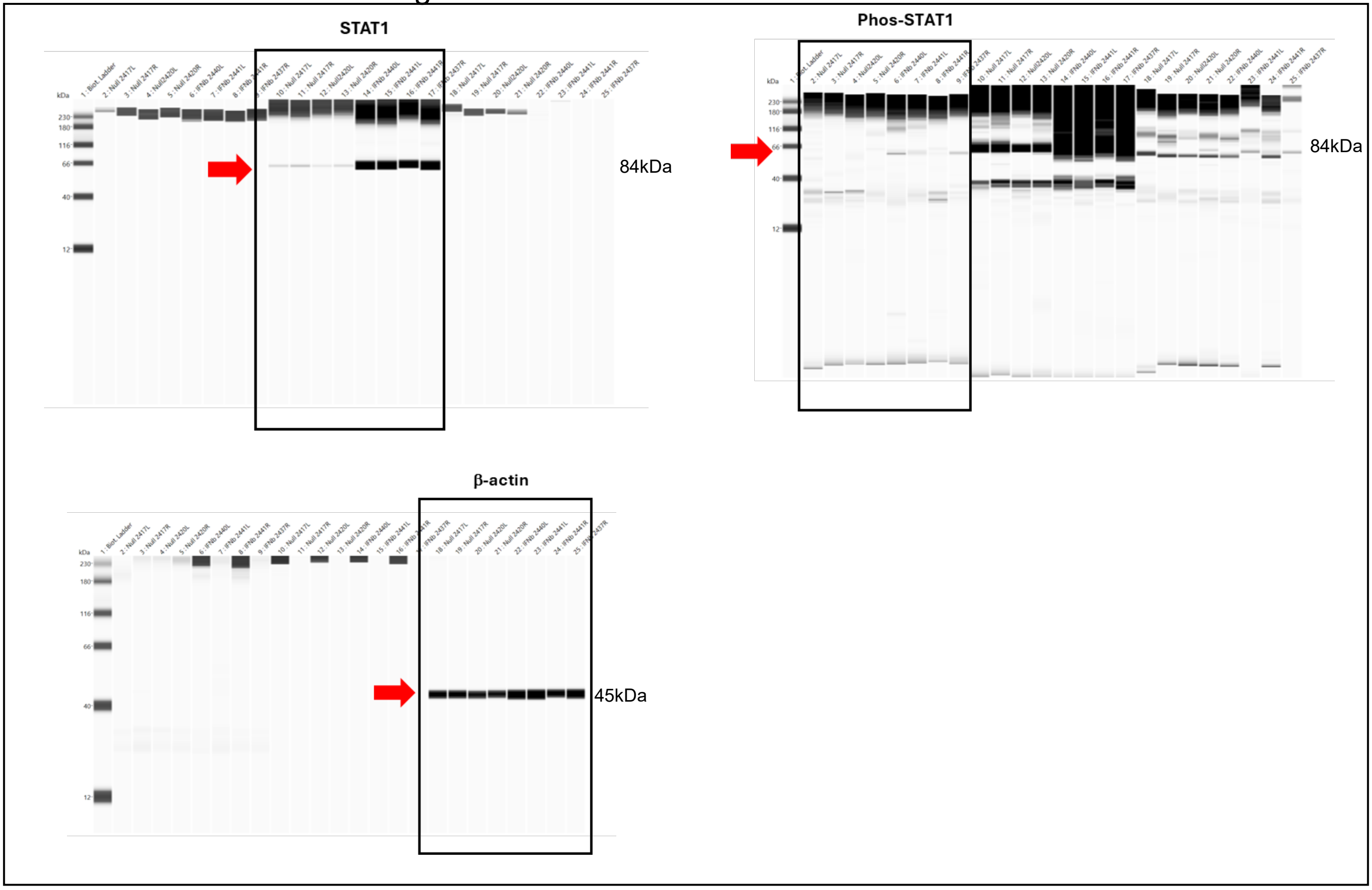

Raw immunoblots for Figure 7h

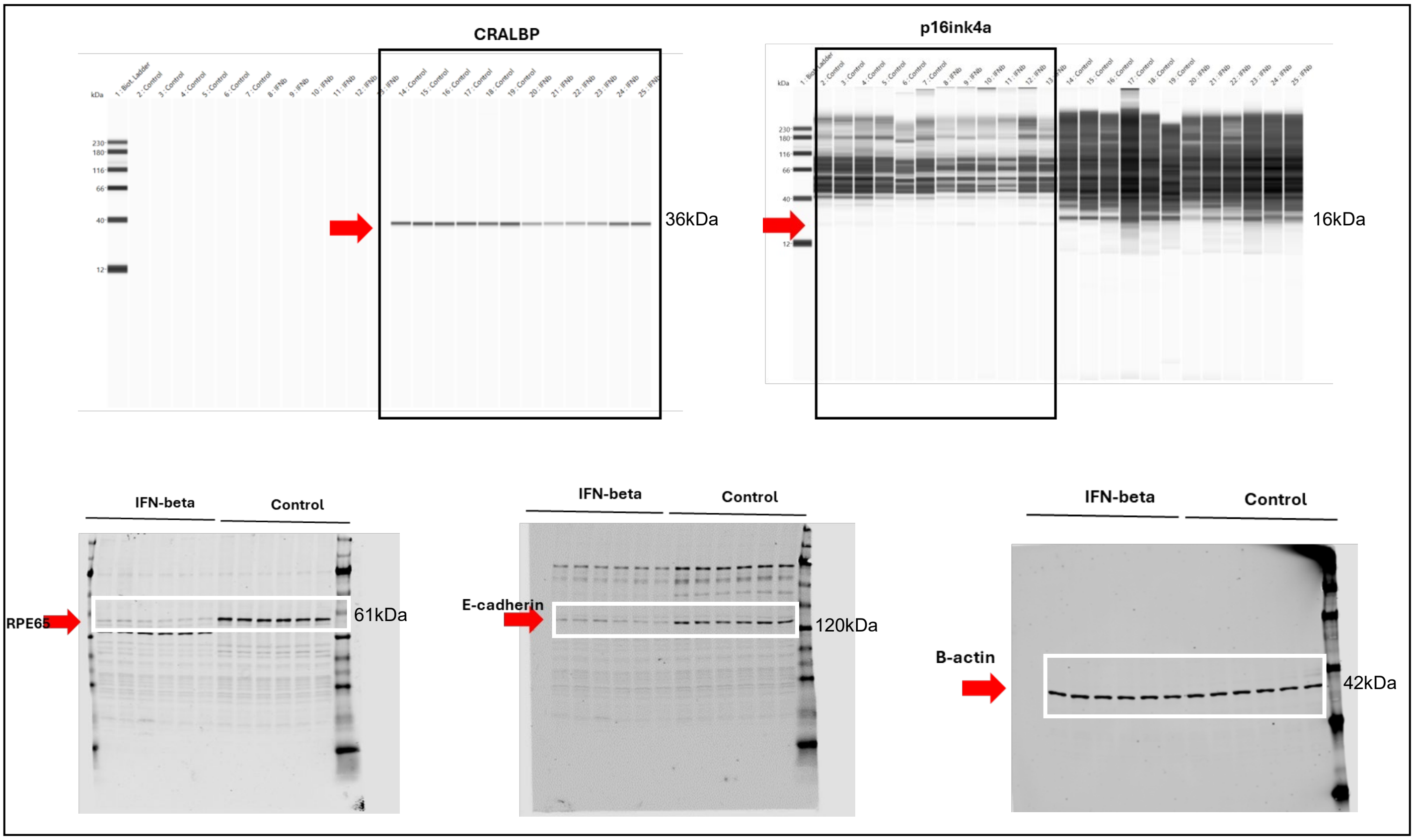

Raw immunoblots for Figure 2l

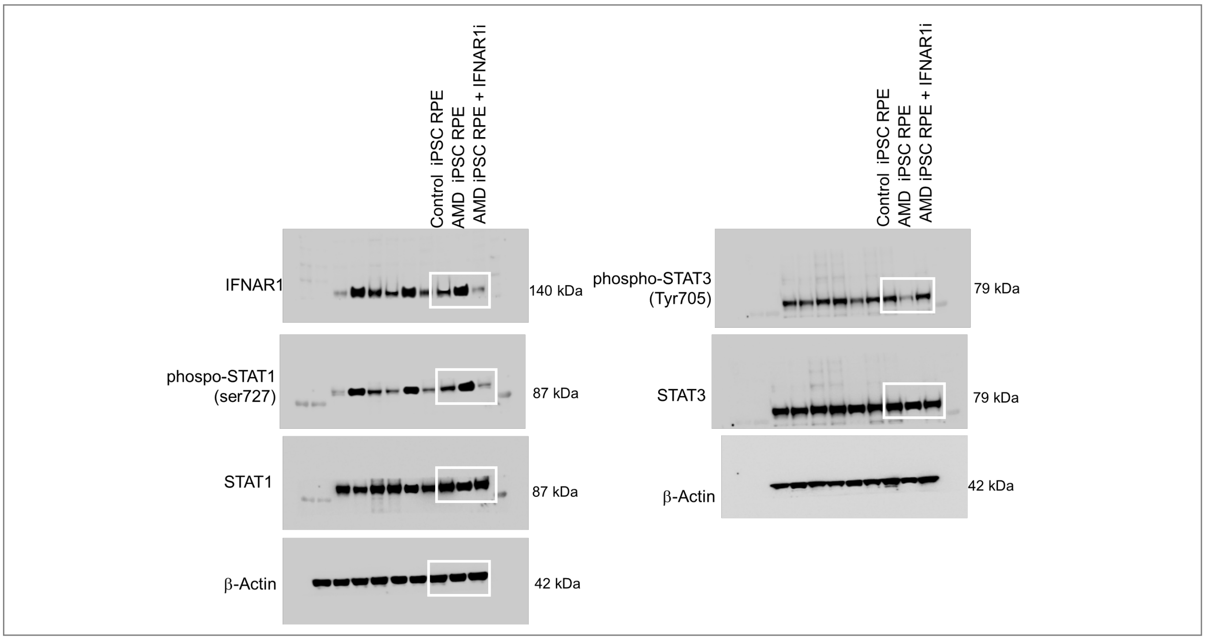

Raw immunoblots for Figure 8j

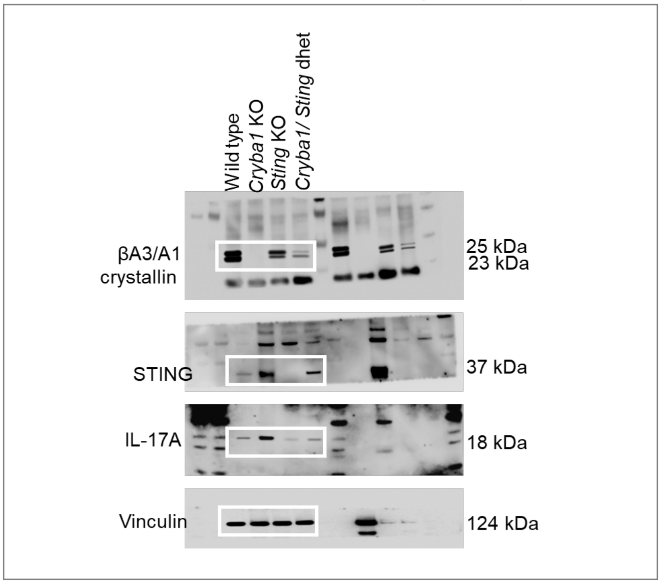

Raw immunoblots for Figure 8l

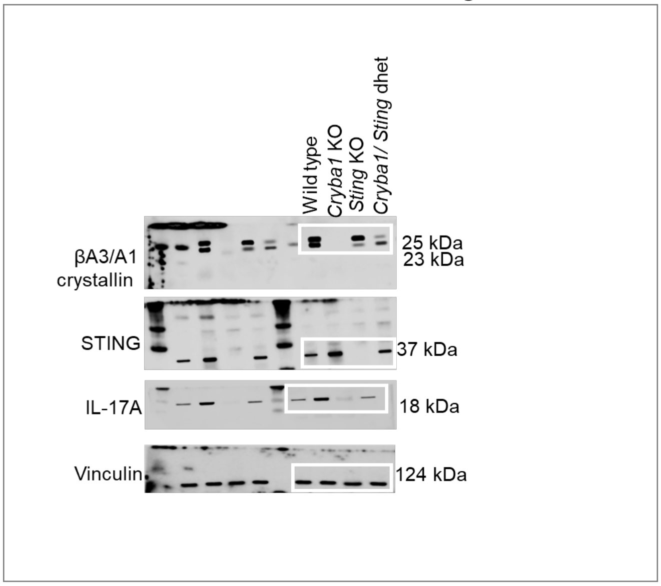

Raw immunoblots of Supplementary Figure 1d

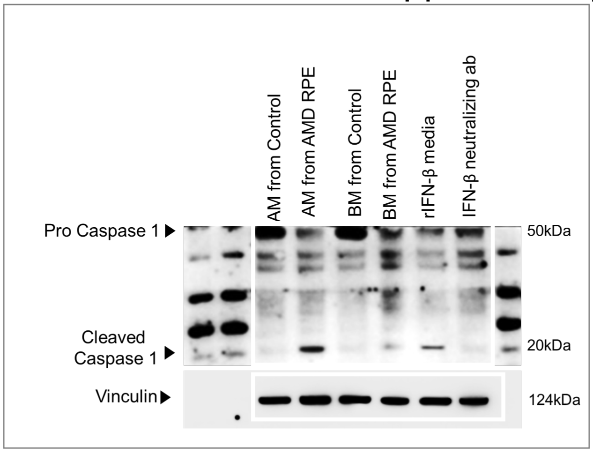

Supplement: Supplementary file 2 — Raw data [file 41419_2026_8491_MOESM2_ESM.pdf]
